# Glucosinolate-Rich Floral Extracts Hinder *Nosema* (= *Vairimorpha*) *ceranae* Infection in Caged *Apis mellifera* Workers

**DOI:** 10.1007/s10886-026-01736-0

**Published:** 2026-07-09

**Authors:** Matteo Carloni, Eleonora Pagnotta, Laura Righetti, Lorena Malaguti, Vittorio Capano, Antonio Nanetti, Nicola Pecchioni, Manuela Bagatta, Luisa Ugolini

**Affiliations:** 1 Department of Life Sciences, University of Modena and Reggio Emilia, Padiglione Besta, Via Amendola 2, Reggio Emilia, 242122 Italy; 2 Consiglio per la Ricerca in Agricoltura e l’Analisi dell’Economia Agraria (CREA), Centro di Ricerca in Cerealicoltura e Colture Industriali, Via di Corticella 133, Bologna, 40128 Italy; 3 Consiglio per la Ricerca in Agricoltura e l’Analisi dell’Economia Agraria (CREA), Centro di Ricerca in Agricoltura e Ambiente, Via di Corticella 133, Bologna, 40128 Italy; 4 Consiglio per la Ricerca in Agricoltura e l’Analisi dell’Economia Agraria (CREA), Centro di Ricerca in Cerealicoltura e Colture Industriali, S.S. 673 metri 25, 200, Foggia, 71122 Italy

**Keywords:** Brassicales, *Eruca sativa*, *Reseda lutea*, Myrosinase, Isothiocyanate, Nosemosis

## Abstract

Colony losses in *Apis mellifera* are caused by multiple stressors, among which the parasitic microsporidian *Nosema* (= *Vairimorpha*) *ceranae*. As an alternative to the controversial fumagillin, sustainable control strategies based on natural bioactive compounds are increasingly being explored. This study evaluated the efficacy of glucosinolate (GSL)-based formulations derived from flowers of *Eruca sativa* and *Reseda lutea* in controlling *N. ceranae* infection in worker honey bees under laboratory conditions. Freeze-dried flowers were characterized for their GSL profile and myrosinase activity, and formulation studies were conducted to choose the best material for bioassays. Then, water-soluble GSL-enriched extracts were produced. Cage feeding assays were performed on uninfected newly emerged bees using sugar syrup supplemented with 2 and 4 µmol g⁻¹ of total GSL of either formulation. Extract administration reduced food consumption and bee survival in a concentration-dependent manner compared to the control group. The lower concentration was further tested on 6-day-old bees artificially infected with *N. ceranae* spores, showing consumption comparable to control, but still a slight decrease in survival. At this concentration, both *E. sativa*- and *R. lutea*-based extracts significantly inhibited *N. ceranae* abundance, by 23.8 and 44.6%, respectively, with no significant difference between plant species. Isothiocyanates—the GSL hydrolysis products—were detected in bee intestines, accumulating primarily in the hindgut and at higher levels in bees fed with *R. lutea* extracts. These findings highlight the potential of floral GSL-rich formulations as natural, environmentally sustainable tools to support colony health and reduce pathogen load.

**Supplementary Information:**

The online version contains supplementary material available at https://doi.org/10.1007/s10886-026-01736-0.

## Introduction

Over the past few years, honey bee (*Apis mellifera* L.) populations have shown periodic declines driven by both biotic and abiotic factors. The microsporidian *Nosema (Vairimorpha) ceranae* is currently one of the most important pathogens affecting adult honey bees and contributing to colony losses. As an obligate intracellular parasite, it is remarkably characterized by temperature plasticity, high spore production, survival capacity (Higes et al. 2010) and adaptation to different hosts (Nanetti et al. 2021a). It infects the epithelial cells of host midgut and leads to digestive disfunction and weakened immune response (Jabal-Uriel et al. 2022). Chronic and asymptomatic infections can weaken colonies by increasing forager mortality, synergically acting with other biotic and abiotic stressors (Singh and Rana 2025). To mitigate the impact of *N. ceranae*, beekeeping practices focusing on improved nutrition, hygienic management, and selective breeding for resistant bee strains are being explored (Formato et al. 2022). The antibiotic fumagillin has been widely used to control *N. ceranae* infections, but concerns about its toxicity, environmental impact and resistance have led to its ban in many countries (Botías et al. 2013). Consequently, natural and sustainable alternative strategies to control *Nosema* spp., including organic compounds, plant extracts and beneficial microorganisms are gaining increasing attention (Iorizzo et al. 2022). The natural compound oxalic acid, used in solution or as component of the commercial product Api-Bioxal^®^, showed efficacy in *N. ceranae* control in both laboratory and field trials (Nanetti et al. 2015; Cilia et al. 2020). In the same study, Cilia et al. also evaluated the commercial product ApiHerb^®^, based on *Allium sativum* and *Cinnamomum zeylanicum* extracts, which successfully reduced *N. ceranae* abundance in honey bee colonies. Probiotics and innovative approaches as RNA interference, are emerging as promising control strategies, especially when combined with plant extracts (Garrido et al. 2024; Tache et al. 2025). Non-thermal atmospheric pressure plasma has also shown antimicrobial potential against *N. ceranae* reducing spore abundance by up to 96% and significantly impairing their viability, cell wall integrity, and infectivity (Boonmee et al. 2025). Natural products and plant-based extracts continue to attract considerable interest: propolis extracts were recently shown to reduce *N. ceranae* infection and enhance honey bee immunity under laboratory conditions (Arredondo et al. 2025) and *Cannabis sativa* (L.) inflorescences, rich in cannabinoids, showed antimicrobial activity against *N. ceranae* in laboratory feeding trials (Fulvio et al. 2025).

Among plants known for their bioactive molecules, the Brassicales represent a large and economically important order characterized by the presence of glucosinolates (GSLs), sulfur-containing metabolites involved in plant defense. Following tissue disruption, GSLs are hydrolysed by the endogenous enzyme myrosinase (MYR), into different biologically active compounds, particularly isothiocyanates (ITCs), the most extensively studied and largely recognized for their antimicrobial activity against soil-borne and human pathogens (Dufour et al. 2015; Romeo et al. 2018), but also nitriles (NITs) and other hydrolysis products depending on reaction conditions and GSL structure (Blažević et al. 2017). MYR-like activity involved in GSL hydrolysis has also been reported in mammalian gut microbiota and have been isolated from Brassica-specialist insects (Beran et al. 2014; Narbad and Rossiter 2018). GSLs vary in profiles among plant organs and are usually most concentrated in seeds. Defatted seed meals in fact have been widely investigated for applications in different agri-food sectors (Pagnotta et al. 2022). Previous studies, conducted at both laboratory and field levels, have shown the activity of *E. sativa* and *Brassica nigra* defatted seed meals, in whitch the single predominant GSL is respectively 4-methylthiobutyl GSL (glucoerucin – GER) and 2-propenyl GSL (sinigrin), in the containment of *N. ceranae* infection, while Borges et al. investigated the use of a pure ITC, sulforaphane (SF), derived from 4-methylsulfinylbutyl GSL or glucoraphanin (GRA) (Borges et al. 2020; Nanetti et al. 2021b). Also, flowers of Brassica *spp*. have recently gained attention for their bioactive content, GSLs, phenolic compounds, and antioxidant activities (Česlová et al. 2023; Okumus 2024). Within the order Brassicales, *E. sativa* (L.) Mill. [*Eruca vesicaria* subsp. *sativa* (Mill.) Hegi], and *Reseda lutea* L., species belonging to the Brassicaceae and Resedaceae families, respectively, are characterized by broad pedoclimatic adaptability, high rusticity and attractiveness to pollinating insects, particularly honey bees (Balfour et al. 2015; Shakeel et al. 2019). Their prolonged flowering periods provide floral resources in Mediterranean areas from spring (*E. sativa*) to late summer (*R. lutea*). *E. sativa* flowers predominantly accumulated GRA and a lower percentage of dimeric 4-mercaptobutyl GSL (dimeric 4-MB GSL) (Bennett et al. 2002; Pagnotta et al. 2025). The prevalent GSL in *R. lutea* flowers is 2-(α-L-rhamnopyranosyloxy)benzyl GSL with minor amounts of benzyl GSL, indol-3-ylmethyl GSL, or glucobrassicin (GBS) and 3-hydroxybenzyl GSL (Radulović et al. 2014; Pagnotta et al. 2020). Building on previous evidence supporting the efficacy of molecules belonging to the GSL family in *N. ceranae* containment and considering the distinctive floral GSL profiles of *E. sativa* and *R. lutea* as well as their ecological roles, the present study investigated floral GSL-rich formulations for the control of *N. ceranae* infection in *A. mellifera ligustica* (Spinola, 1806) under laboratory conditions. Specifically, the aims of the study were to: (i) characterize the GSL-MYR system in *E. sativa* and *R. lutea* flowers; (ii) find the best flower processing strategy to preserve intact GSLs; (iii) produce and formulate GSL-rich extracts for honey bees feeding bioassays; (iv) evaluate bee consumption and tolerance to the formulates; and (v) assess their protective activity in bees artificially infected with *N. ceranae*.

## Methods and Materials

### Plant Material

At the CREA-CI experimental farm in Anzola dell’ Emilia, Bologna, Italy (44°34’30.8"N, 11°09’55.6"E), plots of *E. sativa* and *R. lutea* were sown in two consecutive periods: autumn 2022 and 2023, and spring 2023 and 2024, respectively. This site is in the alluvial plains of the Po Valley, near Bologna in northeastern Italy. Seeds of both plants were sown in three replicated plots. All agronomic practices were applied without chemical inputs (fertilization and pesticides) throughout the vegetative cycle. A homogenous batch of about 1 kg of *E. sativa* flowers was collected in mid-April, while the same quantity of *R. lutea* inflorescences (i.e. the terminal part of the open flowers and buds) was harvested at the end of June. The collected material was immediately frozen at − 20 °C and subsequently freeze-dried using a DLAb 500 (Italian Vacuum Technology). Freeze-dried material was finally ground using a laboratory mill (Retsch ZM200) to a 500 μm grain size, and the powder was stored in a dry environment at room temperature, until processing and analysis.

### Flower Powder Characterization

*Glucosinolate Profile and Content*. GSL analysis was performed by extraction of the freeze-dried flower powder (100–200 mg) with boiling 70% ethanol, followed by enzymatic desulfation and HPLC-UV analysis of desulfated aqueous solution according to ISO 9167:201-1 method (ISO9167-1:1992/Amd 1:2013 1992) with some minor modifications (Pagnotta et al. 2017). A HP 1100 Series Agilent HPLC System (Agilent Technologies, Santa Clara, CA, US) equipped with a Pursuit XRs 5 C18 column (3.0 × 250 mm, 5 μm) and a diode array detector was used for the analysis. The desulfated GSLs were detected by monitoring their absorbance at 229 nm and were identified based on their retention times and UV spectra using an in-house library of purified standards (Franco et al. 2016; Pagnotta et al. 2020, 2025). Sinigrin was used as internal standard and relative response factors were used for quantification (Whatelet et al. 2004). In *R. lutea*, a response factor of 0.5 was adopted for both 3-hydroxybenzyl GSL and O-glycosylated benzyl GSL, according to (Pagnotta et al. 2020). In *E. sativa* a response factor of 1 was adopted for the dimeric 4-MB GSL. Analysis was performed in triplicate and data were expressed as means ± standard deviation (SD).

*Myrosinase Enzyme Activity*. Total MYR activity in the flower powder was determined using the pH-stat method by monitoring the hydrolysis of the sinigrin (0.5 M) in an aqueous 0.1% NaCl solution, maintained at pH 6.5 with 0.01 M NaOH, using a Mettler Toledo DL50 titrator (Finiguerra et al. 2001). One unit of MYR activity was defined as the amount of enzyme that catalyzed the hydrolysis of 1 µmol of sinigrin per min per g of flower powder under the assay conditions.

### Formulation of Glucosinolate-Enriched Feed

*Myrosinase Deactivation and Glucosinolate Autolysis*. Flower powders were treated in an autoclave, at 120 °C and 1 bar for 5, 10–20 min. MYR deactivation was investigated by verifying the GSL autolysis in aqueous buffered solution, performed according to (Pagnotta et al. 2017) with some modifications. 0.1 and 0.2 g of *R. lutea* and *E. sativa* powder were suspended in 2 ml and 5 ml of 50 mM K phosphate buffer, pH 6.5, respectively and kept under agitation for 1 h, at room temperature. The suspension was then extracted with 2 ml of ethyl acetate by vortex agitation. The mixture was centrifuged, and the upper phase was analyzed by GC-MS as follows.

*Qualitative Analysis of Hydrolysis Products by GC-MS and Compounds Tentative Identification*. A Scion GC 436 gas chromatograph coupled with a Scion SQ quadrupole mass detector (Scion Instruments NL BV The Netherlands) and equipped with a ZB-5MSi capillary column (30 m × 0.25 mm i.d., 0.25-µm film; Phenomenex) was used. The oven temperature was set at 60 °C and programmed to rise to 290 °C at 10 °C min^− 1^ and finally hold at 290 °C for 10 min. The transfer line and the ion source were heated at 290 °C and 220 °C respectively. Helium carrier gas had a flow of 1 ml min^− 1^. The split injection mode (1:20) was used. The MS spectrometer operated in electron impact mode at 70 eV, scanning the range of 50/400 m/z, in a full scan acquisition mode. ITC and NIT identification was achieved by comparing MS spectra with the data system library (NIST 11 MS Library), literature data and by comparing the GC retention time and MS spectra with those of pure standard compounds, when available. For GSL hydrolysis products identification, GER ITC standard was produced and purified from the GER GSL as described in (Citi et al. 2019) (> 98% w/w); GER-NIT was produced and purified from the GER GSL as described in (Galletti et al. 2001) with a purity > 95% (w/w); benzyl ITC (BITC, 99.5% purity; Sigma–Aldrich) and benzyl NIT (BNIT, > 99% purity; Sigma-Aldrich) commercial standards were used for comparison of GC and MS characteristics. SF, SF NIT, dimeric 4-MB ITC, 4-(mercapto)butyl ITC (4-MB ITC), 2-(α-L-rhamnopyranosyloxy)BITC, GBS NIT, dimeric 4-MB NIT were tentatively identified by comparison of MS spectra with spectra reported in NIST library and/or literature; 3-hydroxyBITC and 3-hydroxyBNIT MS spectrum were neither present in library (recognized as 4-hydroxyBITC and NIT) nor were found in literature and they were tentatively identified speculating on the original GSL composition of *R. Lutea* flower, and comparing with the spectrum of 3-methoxyBITC and NIT found in NIST library (see *Formulation Tests*, Results section). 2-(α-L-rhamnopyranosyloxy)BNIT was tentatively identified by comparing chromatograms of autolysates in different conditions, since the molecule’s spectrum does not appear in the NIST library or the literature to date, and there are no analytical reference standards available. Spectra correspondence was also confirmed by the machine learning tool Competitive Fragmentation Modeling for Metabolite Identification (CFM-ID 4.0) (Bremer et al. 2022). Data were expressed as percentage of total peak area.

*Additional Investigations for Compound Identification*. Reduction of dimeric 4-MB ITC to 4-MB ITC was performed by adding 5 mg ml^− 1^ of tris(2-carboxyethyl) phosphine hydrochloride (TCEP, Sigma-Aldrich) (Bennett et al. 2002). NITs production was confirmed by autolysis of untreated flowers in the presence of an aqueous solution of 0.1 M FeSO_4_ 0.1 M (Wittstock and Burow 2007).

*Eruca sativa and Reseda lutea Flowers Extract Production*. To produce floral extracts enriched in GSLs, the preparation procedure by Gambari et al. was followed with some modifications (Gambari et al. 2024). The freeze-dried and ground flowers were weighed and gradually added to 30% ethanol (1:5 w/v) at 90 °C under agitation. The mixture was homogenized with an Ultra-Turrax T25 (IKA) instrument for 15 min and cooled in an ice bath. The cooled product was then transferred into 500 ml centrifuge tubes, subjected to sonication for 45 min and followed by centrifugation at 30,100 g at 10 °C, for the same duration. Afterward, the supernatant was paper-filtered, concentrated to 20% of its initial volume using a Rotavapor at 40 °C, and freeze-dried. The obtained powder was homogenized by mortar grinding, hermetically closed in vials and stored at − 20 °C until use. The GSL content of these freeze-dried extracts was determined by solubilizing 40 mg in 10 ml of distilled water and analyzing the solution by desulfation and HPLC-UV analysis as described above.

*Feed Formulation in Sugar Syrup and Glucosinolate Stability.* The freeze-dried extracts from *E. sativa* and *R. lutea* flowers were added, at a concentration of GSLs of 2 and 4 µmol g^− 1^, to the commercial syrup solution Fruttosweet 45 (A.D.E.A. S.r.l., Busto Arsizio, Italy) diluted in water to 58.5% of carbohydrate content. According to the label, the syrup composition was as follows: fructose (45%), glucose (24%), disaccharides (15%), trisaccharides (8%), and other sugars (8%), expressed as a percentage of dry matter. Syrup and extracts were mixed and then sonicated until the extracts were completely dissolved. The solution was then stored in a covered beaker at 33 °C, and its GSL content was checked at the time of preparation and after 3 days. GSL analysis was performed by extracting the syrup in 70% ethanolic solution at 85 °C (2:3 w/v). An aliquot of 1 ml was subjected to the desulfation step and HPLC-UV analysis, as described above. Analysis were performed in triplicate and data were expressed as means ± standard deviation (SD).

### Feeding Trials

*Source of Honey Bees.* The study was conducted in the summer of 2024. Three asymptomatic, fully developed *A. mellifera ligustica* colonies were selected from the experimental apiary of CREA-AA in Bologna (44°31’27.8"N 11°20’57.8"E). Each of them provided two combs of hatching brood, which were incubated for 24 h at 34 °C to allow the emergence of new worker bees.

*Palatability and Tolerability Trial*. Formulated syrups described above were tested in feeding trials to assess food consumption, as an indicator of food palatability, and bee survival. Freshly hatched bees were distributed in plastic cages (10 × 11 × 5.5 cm), with transparent walls and a perforated lid to fit a syringe. About 40 bees were placed in each cage and randomly assigned to 5 groups: control group (C), receiving sugar water solution only, and the other four groups receiving the syrup supplemented with *E. sativa* (E) or *R. lutea* (R) floral extracts at 2 (E2 and R2) or 4 µmol g^− 1^ of total GSLs (E4 and R4). Syrup was distributed in 5 ml needle-free syringes. Three replicates for each treatment were set up in parallel. Then, bees were fed *ad libitum* and incubated at 33 °C throughout the trial, which lasted 15 days. The food was completely replaced every three days. The quantity of food consumed was calculated based on weight difference of the feeders. On the same dates, dead bees were counted and removed from the cages to check for mortality/survival. The average cumulative individual GSL intake was calculated by multiplying the average cumulative food intake by the corresponding GSL concentrations. Living bees were sampled from single cages on day 2 (*n* = 6) and day 6 (*n* = 5) and sacrificed. Their midgut and hindgut were then dissected, promptly frozen into liquid nitrogen, and stored at − 80 °C until ITC analysis.

*Isothiocyanate Analysis in Bee Intestines.* ITC quantification was performed by extraction of intestines with cold methanol followed by the cyclocondensation assay with 1,2-benzenedithiol as described in (Nanetti et al. 2021b). The cyclocondensation product, the 1,3-benzodithiole-2-thione, was analyzed using a Hewlett-Packard chromatograph 1100, equipped with a diode array detector and a Zorbax SB-C18 column (150 × 4.6 mm, 3.5 μm; Agilent Technologies Santa Clara, CA, USA), thermostated at 30 °C. An external calibration curve was generated using methanolic solutions of pure GER ITC and BITC to obtain the cyclocondensation products for ITC content estimation in bees fed with *E. sativa* and *R. lutea* extracts respectively. Results were reported as means ± standard deviation (SD) and expressed as pmol mg^− 1^ of total ITCs in intestines tissues.

### Nosema ceranae Infection Trial

*Honey bee Infection and Experimental Design.* For the *N. ceranae* infection trial, newly emerged bees were transferred into a perforated plastic container, where syringes filled with a 1:1 (w/w) aqueous sugar syrup solution were added and left in an incubator at 33 °C for 5 days, providing food *ad libitum*. *N. ceranae* spores, purified with 95% isotonic Percoll and confirmed by PCR (Urbieta-Magro et al. 2019), kindly provided by the team at the Centre for Apicultural and Agro-Environmental Research in Marchamalo (Spain), were used for the infection of bees. Six-day old bees were used in this trial to ensure correct inoculum assumption during artificial infection (Nanetti et al. 2021b). An aliquot of purified spores was mixed with a 1:1 (w/w) aqueous sugar syrup solution to achieve a concentration of 20,000 spores µl^− 1^. Bees were starved for 2 h, then removed from the incubator and anesthetized with a 40/60% (v/v) air-CO₂ mixture for few minutes. The infection procedure was adapted from (Porrini et al. 2020) with some modifications. Briefly, bees were introduced into the wells of racks for 2 ml tubes, and a perforated plug applied to the open end. The tip of a 10 µl pipette was shortened to facilitate access to the syrup and inserted into the hole. Each bee received 5 µl of the suspension above, corresponding to 100,000 spores. Bees that did not consume the food after one hour were discarded. After inoculation, infected bees were randomly assigned to different cages until the required sample size was reached (*N* = 30) (Nanetti et al. 2021b). The cages were then placed in an incubator at 33 °C and fed with simple sugar syrup for 24 h before treatment administration. Feeding with extracts started one day post-infection (d.p.i.). Treatments included 2 treated groups fed with extract-supplemented syrups at 2 µmol g^− 1^ of GSL for both *E. sativa* and *R. lutea* (E2, R2) and a control group (C) with only syrup. Each treatment was replicated three times in parallel. When possible, five bees per cage were sampled at 3, 6, 8, 10 and 13 d.p.i., immediately frozen in liquid nitrogen and stored at -80 °C for molecular analysis of *N. ceranae* infection. Also, food consumption and tolerability were assessed in the three groups as described above.

*DNA Extraction and qPCR of Nosema ceranae.* Individually collected bee abdomens were homogenized in ultrapure water with a Tissue Lyser II (Qiagen, Hilden, Germany) at 30 Hz with a mixture of glass (425–600 μm) and stainless-steel beads (3 mm) as described by (Cilia et al. 2019). Extraction of total DNA from homogenates was performed using the Quick-DNA Microprep Plus Kit (Zymo Research, Irvine, CA, USA) following the manufacturer’s instructions for solid tissue processing, with minor modifications (Cilia et al. 2018). To quantify the copies of *N. ceranae* genome, a quantitative PCR analysis was carried out on DNAs using primers and probes specific to *N. ceranae Heat-shock protein* 70 (*Hsp 70*) gene following the protocols reported in (Cilia et al. 2018). Quantification of *N. ceranae* DNA copy number was obtained using the standard curve previously constructed by amplifying the *N. ceranae*-specific DNA fragment diluted serially from 1 × 10^0^ to 1 × 10^9^ copies as described in (Ugolini et al. 2021a). All analyses were conducted on two technical replicates.

### Statistical Analysis

Statistical analysis was conducted using R software(R Core Team 2025) considering P ≤ α = 0.05 for all tests. The data on GSL stability in the syrup were analyzed using Student’s *t*-test. Analysis of food consumption was performed applying a repeated measures ANOVA and a linear mixed-effects model using the lme function from the nlme package in R (Pinheiro and Bates 1999). Post-hoc comparisons of time trends across treatments and of food consumption at the final time point (15 days of treatment) were conducted using estimated marginal means and pairwise contrasts with Tukey adjustment for multiple testing (emmeans package) (Searle et al. 1980). The same methodology was applied for GSL intake analysis excluding C group where GSLs were not present. The Kaplan-Meier method was used to estimate the bee survival % in time, and the long-rank/Mantel-Cox test was performed to compare the survival distribution between the groups (survival and survminer packages) (Therneau 2001; Kassambara et al. 2016). The Hazard Ratios (HR) was calculated by the Cox Proportional Hazards model (survival packages). The sampled individuals were considered as right-censored observations. The data on ITC detection in bee guts was analyzed using a factorial ANOVA on log_10_-transformed ITC concentrations to meet normality assumptions (Shapiro-Wilk and Levene’s test). Post-hoc comparisons were conducted as for food consumption. *N. ceranae* number of copies overtime were analyzed using a general linear model on log_10_-transformed number of copies to meet normality assumptions and comparisons among treatment levels were conducted using Newman-Keuls pairwise post-hoc test (Agricolae 2006). *N. ceranae* number of copies was compared at the end of the trials (time 10 dpi) by an ANOVA and post-hoc pairwise comparisons using the Least Significant Difference (LSD) test. The packages dplyr and ggplot2(Wickham et al. 2014; Wickham 2016) were used for data manipulation and visualization, respectively.

## Results

### Characterization of the Glucosinolate-Myrosinase System in *Eruca sativa and Reseda lutea* Flowers

GSL profile and quantitative composition in *E. sativa* and *R. lutea* flowers were investigated. Total GSLs content was 47.2 and 132.0 µmol g^− 1^ for *E. sativa* and *R. lutea*, respectively.

The main compound in *E. sativa* flowers was GRA, 28.2 µmol g^− 1^ (59.7% of total GSLs), followed by dimeric 4-MB GSL, 17.4 µmol g^− 1^ (36.9%) and a small amount of GER, 1.7 µmol g^− 1^ (3.6%). *R. lutea* flowers contained more than twice the total content of GSLs than *E. sativa*, mainly composed of 2-(α-L- rhamnopyranosyloxy)benzyl GSL, 100.6 µmol g^− 1^ (76.2%) with comparable proportions of benzyl GSL, 12.1 µmoli g^− 1^ (9.2%), GBS, 8.6 µmol g^− 1^ (6.5%), and 3-hydroxybenzyl GSL, 10.6 µmol g^− 1^ (8.0%). Representative HPLC-UV chromatograms of *E. sativa* and of *R. lutea* flower desulfated GSLs is reported in Supplemental materials Fig. S1.

MYR activity was detected in both flower powders, but *R. lutea* showed substantially higher activity, 23.4 ± 0.6 U, compared to *E. sativa*, 5.9 ± 0.7 U.

### Formulation Tests

*Flower Deactivation of Myrosinase: Impact on Glucosinolate and Glucosinolate Hydrolysis Product Profiles*. To explore the possibility of using intact flower powders for formulation development while preventing GSL hydrolysis, MYR deactivation tests were conducted by treating flowers in autoclave at 120 °C and 1 bar, for different times (5-10-20 min). GSLs in treated *E. sativa* and *E. lutea* flowers were quantified and residual GSL % is shown in Tables 1 and 2 respectively.

**Table 1:** Residual Glucosinolate (GSL) % of *Eruca sativa* freeze dried flowers after treatment in autoclave at 120 °C and 1 bar for 5-10-20 min

GSL	5 min (%)	10 min (%)	20 min (%)
GRA	32.6	13.5	2.8
GER	29.4	35.3	11.8
dimeric 4 MB GLS	31.0	13.2	0.0
Total	32.0	14.2	2.1

**Table 2:** Residual Glucosinolate (GSL) % of *Reseda lutea* freeze dried flowers after treatment in autoclave at 120 °C and 1 bar for 5-10-20 min

GSL	5 min (%)	10 in (%)	20 min (%)
Benzyl GSL	34.7	26	30.7
2-(α-rhamnosyloxy)benzyl) GSL	67.1	47.5	43.3
GBS	25.6	19.8	5.9
3-OH-benzyl	74.5	57.6	33.0
Total	61.2	43.9	39.2

Autoclave treatment determined a consistent loss in total GSLs in *E. sativa* flowers, which decreased to 15.1 µmol g^− 1^ as soon as after 5 min of treatment (− 68.0%) and became more severe over time. The treatment affected single GSLs in a similar way (about 30% loss in 5 min of teatment). In *R. lutea*, autoclave treatment was less detrimental than in *E. sativa*, nevertheless it determined a decrease of total GSL content to 81.7 and 58.7 µmol g^− 1^, in 5 and 10 min respectively (-38.8 and 56.1%), more marked for the Benzyl GSL and the GBS (about 70% loss in 5 min of treatment).

To verify the extent of MYR deactivation by autoclave treatment and its effect on GSL hydrolysis, autolysis of the flower powders, before and after thermal deactivation, was carried out and possible GSL degradation products were qualitative detected by GC-MS analysis. Autolysis was performed under different conditions and results obtained from *E. sativa* are reported in Table 3.

**Table 3:** GSL hydrolysis products formed by *Eruca sativa* flowers autolysis conducted under different conditions and analyzed by GC-MS. Autolysis was performed on flower powders: (1) as such; (2) in presence of tris(2-carboxyethyl) phosphine hydrochloride (TCEP); (3) deactivated by autoclave treatment (5 min at 120 °C and 1 bar); (4) in presence of FeSO_4_. % area of the compound peaks is reported. Glucoerucin (GER); sulforaphane (SF); 4-(mercaptobutyl)isothiocyanate (4-MB ITC), 4-(mercaptobutyl)nitrile (4-MB NIT), bis(4-isothiocyanatobutyl)disulfide (dimeric 4-MB ITC), bis(cyanatobutyl)disulfide (dimeric 4-MB NIT), isothiocyanate (ITC), nitrile (NIT)

	Autolysis conditions (% peak area)			
Compounds	Non- deactivated flowers	Non- deactivated flowers + TCEP	Deactivated flowers, 5 min	Non- deactivated flowers + FeSO_4_
Dimeric 4-MB ITC	46.0	13.4	-	-
SF	25.5	4.25	-	-
GER ITC	2.57	3.16	-	-
4-MB ITC	-	16.34	-	-
Dimeric 4-MB NIT	-	-	16.06	12.88
SF NIT	-	-	1.30	0.82
GER NIT	-	-	4.8	2.39

Compound peak GC retention time (GC t_R_), the mass spectra (MS) peak composition and the identification method (ID) are reported in Supplemental materials Table S1, while GC chromatograms and MS spectra are reported in Supplemental Fig. S2-S3.

The GC chromatogram of the autolysis products formed from the thermally untreated *E. sativa* flowers showed the dimeric 4-MB ITC, derived from the corresponding dimeric 4-MB GSL, as the main compound. SF, the ITC derived from GRA, was detected, with a smaller peak area in comparison to the dimeric 4-MB ITC. A small peak of GER ITC, derived from GER, was also found (Table 3, Supplemental Fig. S2 A – S3). The autolysis of *E. sativa* flowers in the presence of the reducing agent TCEP brought to the detection of a new peak (t_R_ 9.9 min), with mass spectrum corresponding to the previously reported spectrum of the monomeric 4-MB ITC, derived from the dimeric 4-MB GSL(Cerny et al. 1996; Chiang et al. 1998; Bennett et al. 2002; Raffo et al. 2018; Fechner et al. 2018) (Table 1, Supplemental Fig. S2 B – S3). No 4-MB ITC was found in the autolysis of unprocessed *E. sativa* flowers, as expected from the GSL profile of the flowers, which showed the presence only of the dimeric 4-MB GSL (Table 1). When *E. sativa* flowers had been treated by autoclave, no ITCs, but NITs were found, with the prevalence of the Dimeric 4-MB NIT (Table 3, Supplemental Fig. S2 C – S3). Autolysis on thermally untreated *E. sativa* flowers, in the presence of FeSO_4_, led to the production of NITs with a similar trend. Indeed, GER NIT was identified by comparison with a pure standard while SF NIT and dimeric 4-MB NIT were putatively identified based on new peak occurrence evaluation comparing chromatograms obtained from autolysis in presence or absence of FeSO_4_ and literature data (Table 3, Supplemental Fig. S2 D – S3). No ITCs were found.

Results obtained by *R. lutea* autolysis experiments are reported in Table 4.

**Table 4:** GSL hydrolysis products formed by *Reseda lutea* flowers autolysis conducted under different conditions and analyzed by GC-MS. Autolysis was performed on flower powders: (1) as such; (2) and (3) deactivated by autoclave treatment for 5 and 10 min at 120 °C and 1 bar, respectively; (4) in presence of FeSO_4_. % area of the compound peaks is reported. Benzyl (B), isothiocyanate (ITC), nitrile (NIT), indol-3-ylmethyl- (GBS)

	Autolysis conditions (% peak area)			
Compounds	Non- deactivated flowers	Deactivated flowers, 5 min	Deactivated flowers, 10 min	Non- deactivated flowers + FeSO_4_
2-(α-L- rhamnopyranosyloxyl)BITC	44.85	18.6	-	22.93
BITC	6.52	-	-	-
3-hydroxyBITC	2.51	-	-	-
2-(α-L- rhamnopyranosyloxyl)BNIT	-	26.44	8.84	34.82
BNIT	-	3.36	5.48	6.85
3-hydroxyBNIT	-	11.80	5.83	7.38
GBS NIT	-	3.64	2.32	4.92

Compound peak GC retention time (GC t_R_), the mass spectra (MS) peak composition and the identification method (ID) are reported in Supplemental materials Table S2, while GC chromatograms and MS spectra are reported in Supplemental Fig. S4-S5.

The GC chromatogram from the non-thermally deactivated *R. lutea* flower autolysis indicated that 2-(α-L-rhamnopyranosyloxy)BITC was the main peak, as the corresponding GSL is prevalent in the flower GSL profile (Radulović et al. 2014). BITC, the hydrolysis product of benzyl GSL, was also found (Table 4, Supplemental Fig. S4 A – S5). The MS spectrum of the hydrolysis product 3-hydroxyBITC was neither present in NIST library nor reported in literature. One chromatogram peak (t_R_ 14.1 min) was tentatively assigned to 3-hydroxyBITC, based on the MS spectrum of 3-methoxy BITC available in the NIST library, whose main fragments corresponded to the same main fragments plus 14 u of the additional methyl group. CFM-ID 4.0 predicted spectrum also confirmed the structure (Bremer et al. 2022). The GBS ITC was not detected. Similarly to *E. sativa*, in *R. lutea* flower powders subjected to autoclave treatment for 10 min and autolysis, NITs were produced. BNIT, presence was confirmed by the authentic standard, and GBS NIT was recognized by NIST library and compared with literature data (Table 4, Supplemental Fig. S4 B and C – S5). The 2-(α-L-rhamnopyranosyloxy)BNIT and 3-hydroxyBNIT MS spectra were neither present in NIST library nor reported in literature and were tentatively identified by comparing peak chromatograms of treated and untreated samples and CFM-ID 4.0 predicted spectra (Bremer et al. 2022) (Table 4, Supplemental Fig. S4 B and C– S5). 3-hydroxyBNIT was also assigned referring to the 3-methoxy BNIT spectrum available in the NIST library, as for the 3-hydroxyBITC (see above). The autolysis of non-deactivated *R. lutea* flowers in presence of FeSO_4_ led to the production of NITs (Table 4, Supplemental Fig. S4 D – S5). Differently from *E. sativa* flowers, autolysis of 5 min-treated *R. lutea* flowers resulted not only in the detection of NITs, but also in the production of 2-(α-L-rhamnopyranosyloxy)BITC (Table 4, Supplemental Fig. S4 B).

*Glucosinolate-Enriched Extract Production from Eruca sativa and Reseda lutea Flowers and Formulation in Sugar Syrup.* Due to the loss of GSLs and NITs production during autoclave flower deactivation and to overcome GSL hydrolysis by MYR in formulation development, an extract enriched in GSLs was produced by hot ethanolic extraction from flower powder. *E. sativa* and *R. lutea* yield of total GSL extraction was 56.5% and 45.8% of the GSL flower content, respectively. Results of qualitative and quantitative analysis of the GSL content in the flower extract are shown in Tables 5 and 6 for *E. sativa* and *R. lutea* respectively.

**Table 5:** Glucosinolate (GSL) content (µmol g^− 1^) of *Eruca sativa* freeze dried flower extract. 4-methylsulfinylbutyl GSL (GRA); 4-methylthiobutyl GSL (GER); dimeric 4-mercaptobutyl GSL (dimeric 4-MB GSL)

GSL	(µmol g^− 1^)
GRA	74.9 ± 2.2
GER	3.2 ± 0.1
Dimeric 4-MB GSL	27.1 ± 0.5
Total	105.2 ± 1.8

**Table 6:** Glucosinolate (GSL) content (µmol g^− 1^) of *Reseda lutea* freeze dried flower extracts. Indol-3-ymethyl GSL (GBS)

GSL	(µmol g^− 1^)
Benzyl GSL	7.1 ± 0.1
2-(α-L- rhamnopyranosyloxyl) benzyl GSL	161.0 ± 2.1
GBS	11.3 ± 1.5
3-hydroxybenzyl GSL	19.7 ± 0.1
Total	199.2 ± 3.0

**Fig. 1:**
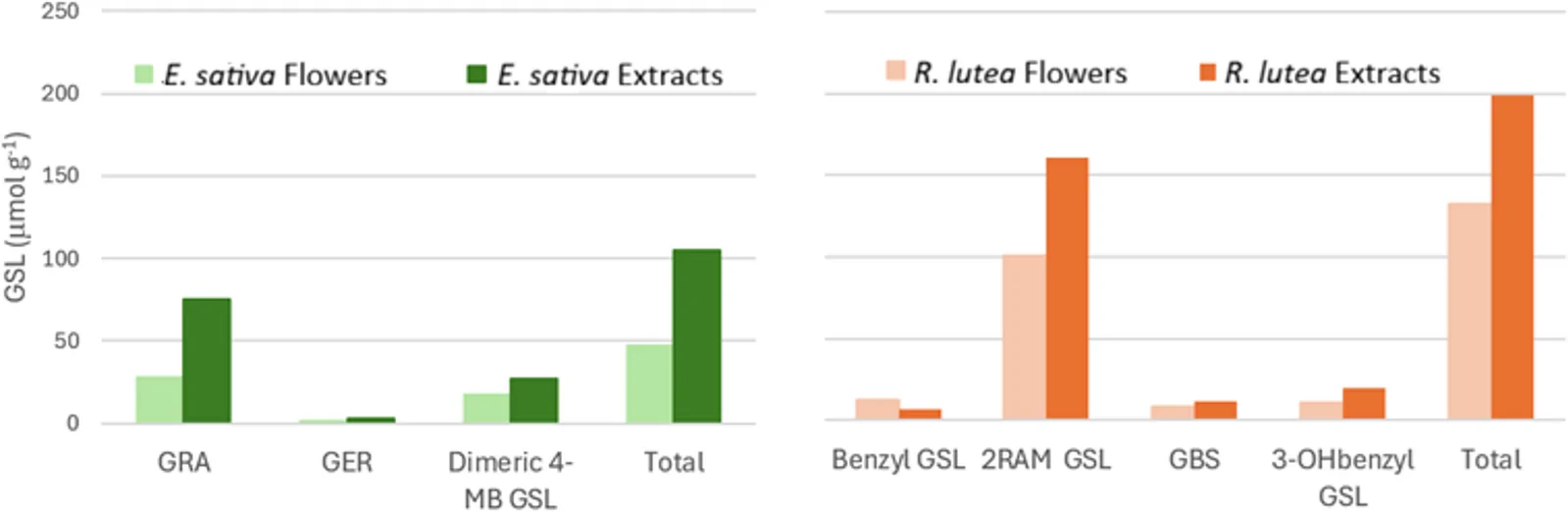
GSL content (µmol g^− 1^) and composition of *Eruca sativa* and *Reseda lutea* flowers and extracts. 2-(α-L- rhamnopyranosyloxy)benzyl GSL (2RAM GSL); 3-hydroxybenzyl GSL (3-OHbenzyl GSL)

The GSL content in the extract was more than double that of the intact flowers in *E. sativa*, whereas it was slightly less than doubled in *R. lutea*. Compared to the composition of *E. sativa* flowers, the extract had a higher % of GRA (71% vs. 60%) and a lower % of dimeric 4-MB GSL (26% vs. 37%). In *R. lutea*, the GSL % composition in the extract did not differ substantially from that of the flowers. A comparative summary plot of GSL content and composition in *E. sativa* and *R. lutea* flowers and extracts is shown Fig. 1.

Extracts were solubilized in diluted sugar syrup (Fruttosweet^®^) at 2 and 4 µmol g^− 1^ of total GSLs and GSL stability after three days of storage at 33 °C was verified. No statistical evidence (*P* < 0.05) of GSL degradation was observed within the 3 days observation period (Supplemental Table S3 and S4 for *E. sativa* and *R. lutea* extracts respectively).

### Palatability and Tolerability Trial

*Food and Glucosinolate Consumption.* Syrup formulations with extracts at two GSL concentrations (2 and 4 µmol g^− 1^) were evaluated for their palatability, reflected by food consumption, and tolerability to new-born worker honey bees.

**Fig. 2:**
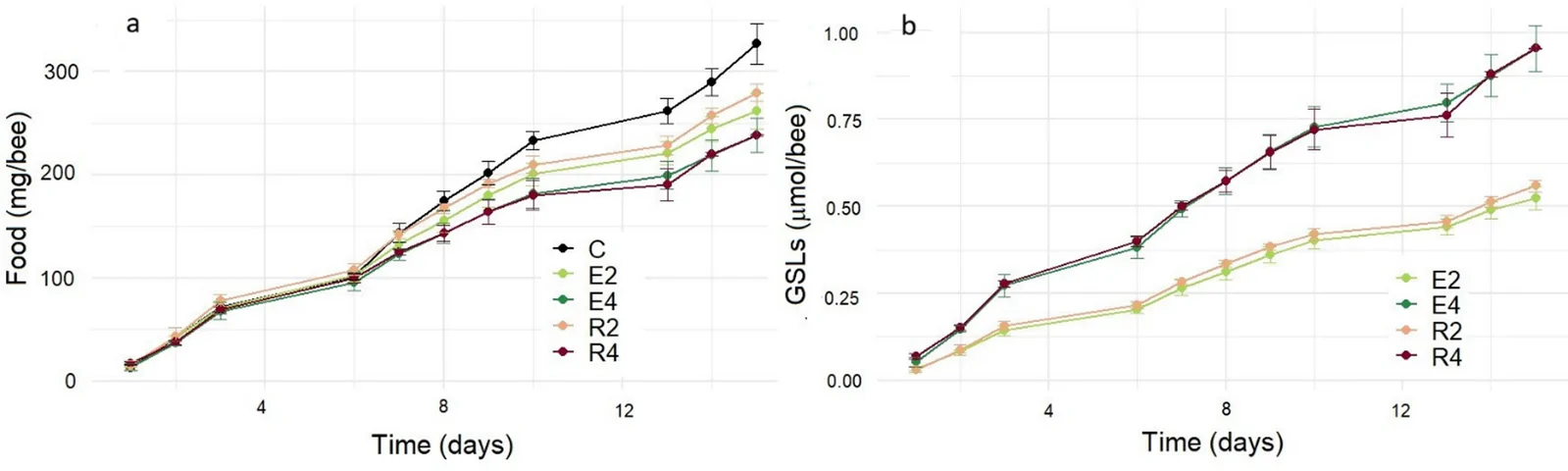
Cumulative individual food consumption of bees fed with different kinds of food, expressed as mg/bee (**a**), and relative cumulative individual GSL intake, expressed as µmol/bee (**b**). Vertical bars indicate standard deviation. *Eruca sativa* and *Reseda lutea* extracts at 2 and 4 µmol g^− 1^ of GSL (E2-E4, R2-R4); control with sugar syrup (C)

Results reporting individual bee cumulative food intake are shown in Fig. 2, a.

Food consumption significantly increased with time (*F*(1,153) = 5469.117, *P* < 0.001), as expected, and the kind of food significantly influenced the individual food consumption (*F*(4,153) = 37.042, *P* < 0.001). The effect of different foods changed with time (*F*(4,153) = 24.769, *P* < 0.001). Pairwise comparisons of the slopes revealed that: C had a significantly steeper increase in consumption over time compared to all other treatments (E2, E4, R2, R4, *P* < 0.001); R2 consumption was significantly greater than that for R4 and E4 (*P* < 0.05); E2 had higher values than R4 (*P* < 0.05); there was no statistical difference between E2 vs. R2 and E4, or E4 vs. R4. Taking into account only the final point of the assay, at 15 days of treatment, the Tukey HSD post-hoc results showed that total food consumption in group C (337.4 mg/bee) was significantly higher than all treated groups, R4 and E4 consumption (246.2 and 231.8 mg/bee respectively) were lower than R2 (279.7 mg/bee) (*P* < 0.05), while no difference was found between E2 (265.6 mg/bee) and E4, R2 and R4, nor between E4 and R4.

Considering GSL intake (Fig. 2, b) there were significant effects for treatment (*F*(3,122) = 344.56, *P* < 0.001), time (*F*(1,122) = 3580.50, *P* < 0.001), and their interaction (*F*(3,122) = 86.25, *P* < 0.001). Tukey post-hoc pairwise comparisons among treatment groups revealed in both the analyses of GSL intake over time and at the final point no statistical difference between treatments at the same concentrations, while both E2 and R2 showed significantly lower values compared to E4 and R4 (*P* < 0.001). Although food consumption decreased at higher concentrations, GSL intake in E4 and R4 still exceeded that in E2 and R2.

**Fig. 3:**
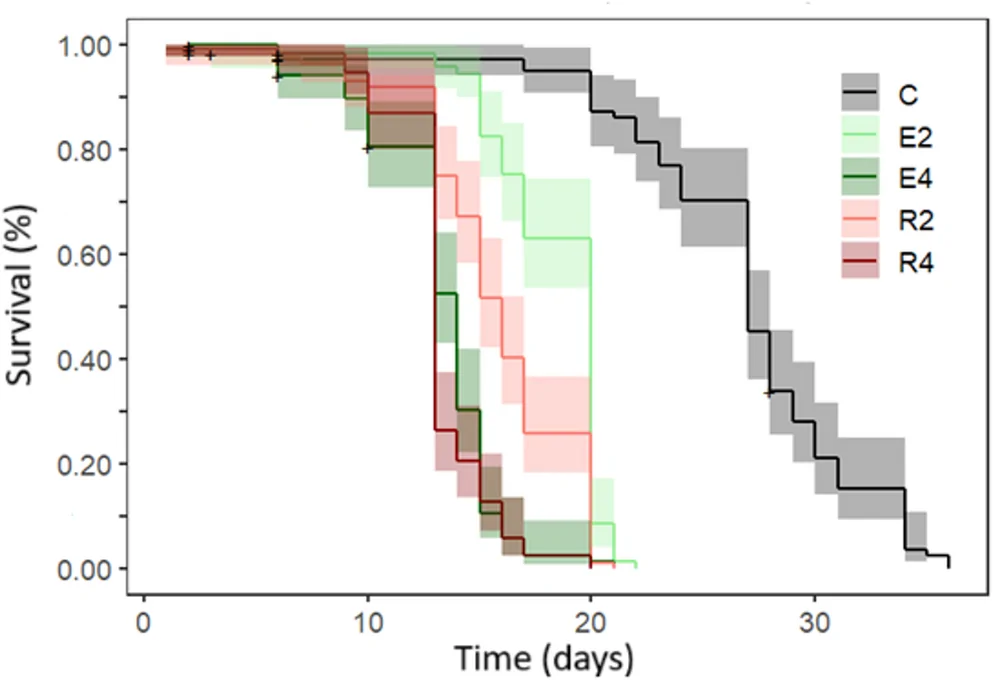
Kaplan-Meier survival curves for bees of control (C) and treated groups at 2 (E2, R2) and 4 (E4, R4) µmol g^− 1^ of GSLs concentration in sugar syrup. Crosses indicate right-censored cases. The solid lines represent the estimated survival probabilities (%) over time; the shaded areas indicate the 95% confidence intervals for each group

*Bee Survival.* The Kaplan-Meier method was used to estimate the percentage (%) of bees that survived over time (days of feeding) for the five groups and the obtained curves are reported in Fig. 3.

Overall, the Cox regression model indicated that the kind of treatment had a significant effect on bee survival (*Score log-rank test* = 417.7 on 4 df, *P* < 0.001).

The pairwise test showed a significant difference between survival of C and treated groups at both concentrations (C-E2, *score log-rank test* = 149.9 on 1 df, *P* < 0.001; C-R2 *score log-rank test* = 172.4 on 1 df, *P* < 0.001; C-E4 *score log-rank test* = 188.2 on 1 df, *P* < 0.001; C-R4 *score log-rank test* = 189.9 on 1 df, *P* < 0.001). There was a significant difference also between the two concentration curves of the same extracts (E2-E4, *score log-rank test* = 101.6 on 1 df, *P* < 0.001; R2-R4, *score log-rank test* = 45.09 on 1 df, *P* < 0.001) and between the lower concentrations of the two different extracts (E2-R2, *score log-rank test* = 23.58 on 1 df, *P* < 0.001), where the R2 extract had a higher impact on bee survival compared to E2, at the lower concentration. Instead, at the highest concentration there was no significant difference between the bee survival of E4 and R4 groups.

The restricted mean survival time calculated for the individual groups is reported in Supplemental materials (Supplemental Table S5).

The ratio of hazards (HR) between the hazard rate of treated groups and control, was also determined (Table 7).

**Table 7:** Hazard ratio (HR) confidence interval (CI)

Groups	HR	95% CI	*P*-value
C	—	—	
E2	18.4	10.0, 33.7	< 0.001
E4	87.2	46.5, 163	< 0.001
R2	35.1	18.9, 65.2	< 0.001
R4	109	57.5, 205	< 0.001

The HR > 1 shows how treatments E and R, at any concentration, significantly increased hazard of death of bees compared to C.

**Fig. 4:**
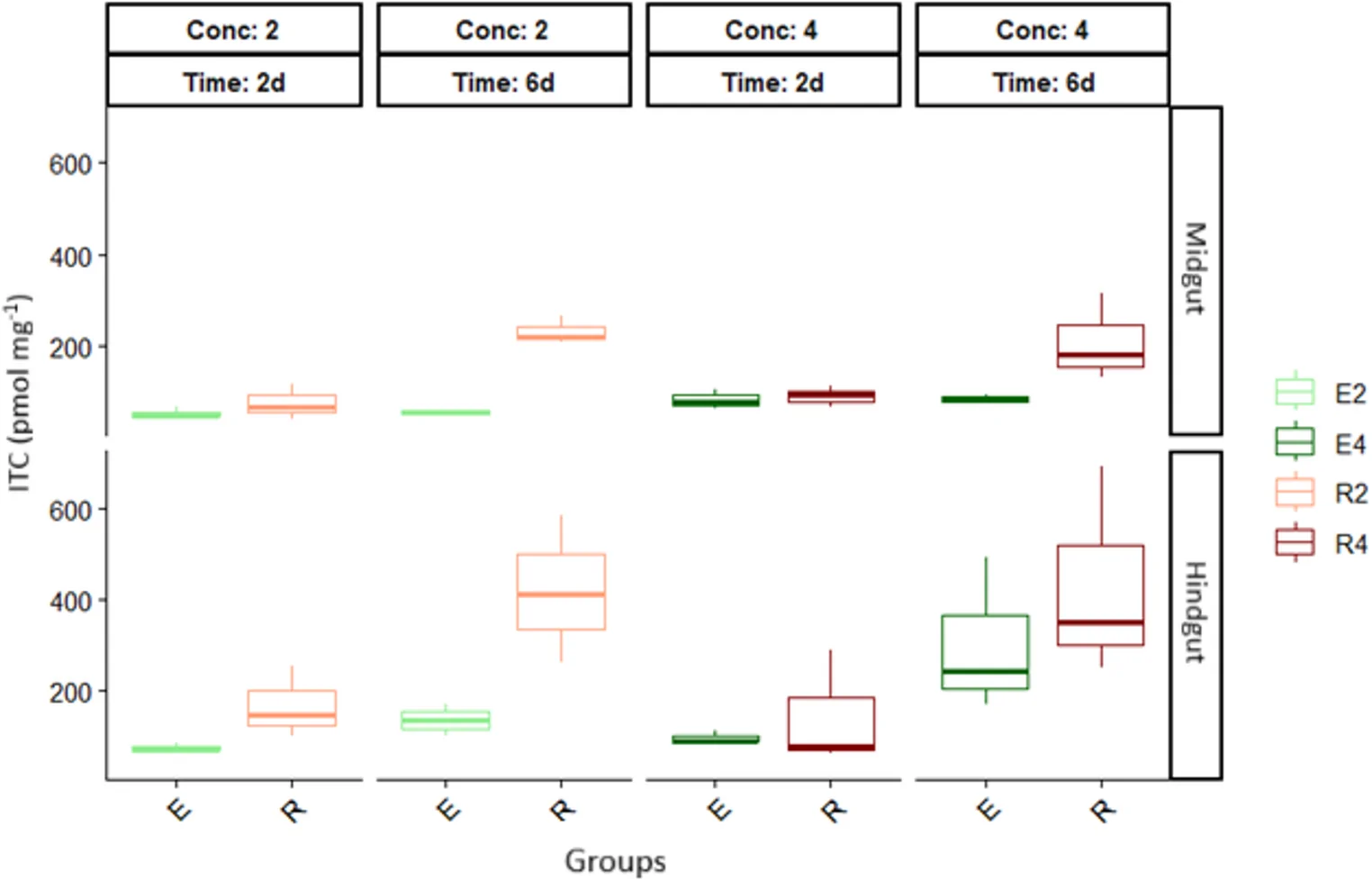
Boxplots showing the distribution of ITC concentrations (pmol mg^− 1^) in bee gut tissues (midgut and hindgut) treated with products, *Eruca sativa* and *Reseda lutea* flower extracts, E and R, at two concentrations (2 and 4 µmol g^− 1^) and sampled at two time points (2 and 6 days). The horizontal line inside each box represents the median value, the lower and upper edges of the box indicate the first and third quartiles (interquartile range, IQR), and the whiskers extend to 1.5 × IQR from the quartiles

*Isothiocyanate Detection in Gut.* ITCs and possible ITC-adducts were found in the midgut and hindgut of both treated groups as early as two days after extracts administration (Fig. 4).

The ANOVA on log-transformed ITC values revealed that treatments (*F*(1, 32) = 31.09, *P* < 0.001), gut region (*F*(1, 32) = 31.50, *P* < 0.001) and time (*F*(1, 32) = 43.00, *P* < 0.001) significantly influenced ITC concentration, while the effect of the extract concentration was not significant. Moreover, the effect of the treatment changed with the extract concentration (*F*(1, 32) = 5.19, P = < 0.05) and time (*F*(1, 32) = 7.95, *P* < 0.05). R groups generally showed higher ITC values compared to E groups, the hindgut group displayed higher ITC values than midgut and ITC accumulated with time. Results revealed that ITC concentrations in the hindgut of bees fed with *R. lutea* and sampled at 6 days exhibited the highest values. Conversely, bees treated with *E. sativa* and sampled at 2 days exhibited, in the midgut, the lowest values. ITC analysis was performed in control bee intestines too, but no ITC was found. Considering the GSL intake recorded during the 24 h prior to sampling and the ITC amounts quantified in the entire gut (hindgut + midgut), the calculated GSL-ITC conversion rates were 3.6% and 4.1% for the E2 and E4 samples, and 12.4% and 7.5% for the R2 and R4 in samples after 2 days of feeding. After 6 days 3.2 and 3.5% of the ingested GSLs were converted in ITCs for E2 and E4, and 11.2% and 5.4% for R2 and R4 respectively.

### *Nosema ceranae Trial*

The lowest concentration tested during the food consumption and tolerance trials (2 µmol g^− 1^ of GSL) was selected to evaluate the effect of the extracts on *N. ceranae* bee infection. Treatment began at 1 d.p.i. and food consumption, and tolerability evaluations were repeated at the assay conditions.

**Fig. 5:**
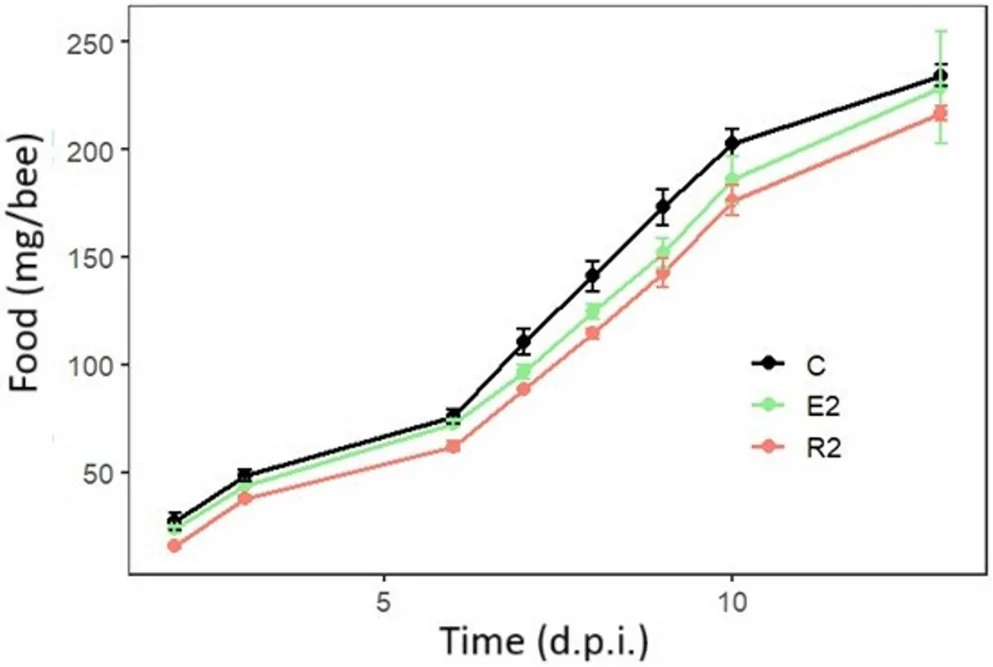
Cumulative individual food consumption overtime (days post infection, d.p.i.) of bees fed with different kinds of food (C, E2, R2), expressed as mg/bee. Vertical bars indicate standard deviation. *Eruca sativa* and *Reseda lutea* extracts at 2 µmol g^− 1^ of GSL (E2 and R2); control with sugar syrup (C)

*Food Consumption.* Bee cumulative food consumption recorded for the three treatment groups is reported in Fig. 5.

The repeated measures ANOVA did not show any significant effects of treatments on cumulative food consumption nor on treatment × time interaction, suggesting that differences among treatments did not vary over time. As expected, a significant effect of time was observed (*F*(7, 42) = 431.44, *P* < 0.001), indicating that consumption increased over the measurement period.

At the end of the trial, at 13 d.p.i., mean individual cumulative consumption was 234.42 ± 8.45 mg/bee (mean ± SD) for C, 229.12 ± 45.07 mg/bee for E2, and 216.75 ± 6.10 mg/bee for group R2. No significant differences between groups were detected. GSL intake trend reflected those of food consumption as GSL were tested at a single concentration. Cumulative GLS intake at 13 d.p.i. was 0.46 and 0.43 µmol/bee for E2 and R2 respectively.

**Fig. 6:**
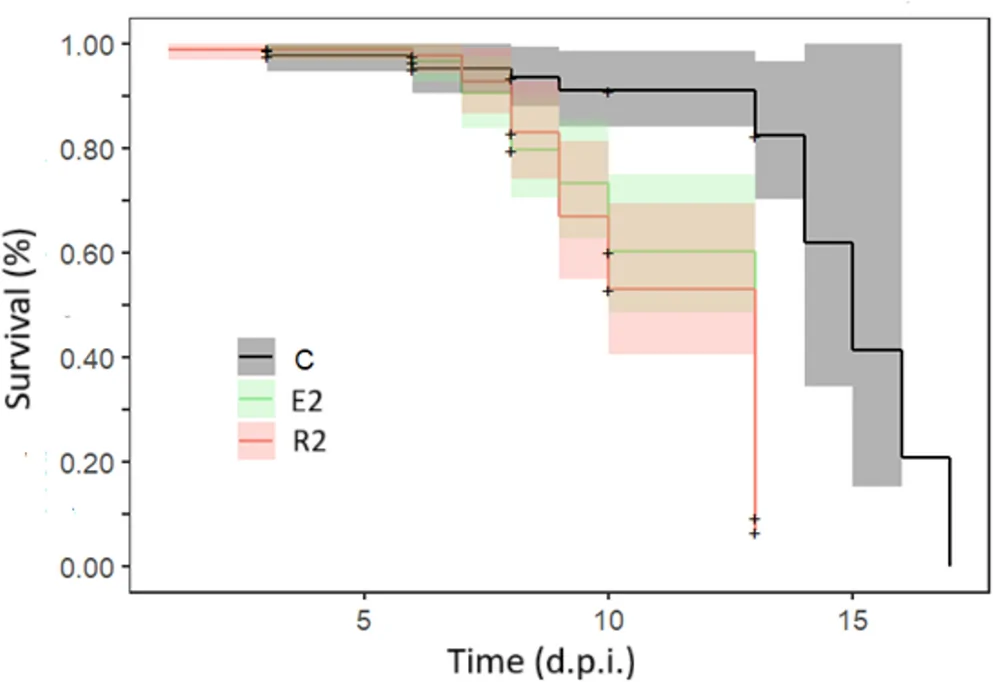
Kaplan-Meier curves for infected bees of control (C) and treated groups (E2, R2). Crosses indicate right-censored cases. The solid lines represent the estimated survival probabilities (%) over time; the shaded areas indicate the 95% confidence intervals for each group

*Bee Survival.* Treatments influenced bee survival as described in Fig. 6.

Overall, the Cox regression model indicated that the kind of food had a significant effect on bee survival (*Score log-rank test* = 33.3 on 2 df, *P* < 0.001).

The pairwise test showed a significant difference between C and both E2 (Score log-rank test = 26.76 on 1 df, *P* < 0.001) and R2 (*Score log-rank test* = 26.31 on 1 df, *P* < 0.001) survival, while there was no significant difference between the two treatments E2 and R2.

The restricted mean survival time calculated for the individual groups is reported in Supplemental materials (Supplemental Table S6).

Hazard ratio (HR) was calculated and showed that E2 and R2 had a significantly higher hazard of death than C (Table 8).

**Table 8:** Hazard ratio (HR), confidence interval (CI)

Groups	HR	95% CI	*P*-value
C	—	—	
E2	6.84	3.11, 15.0	< 0.001
R2	7.49	3.36, 16.7	< 0.001

**Fig. 7:**
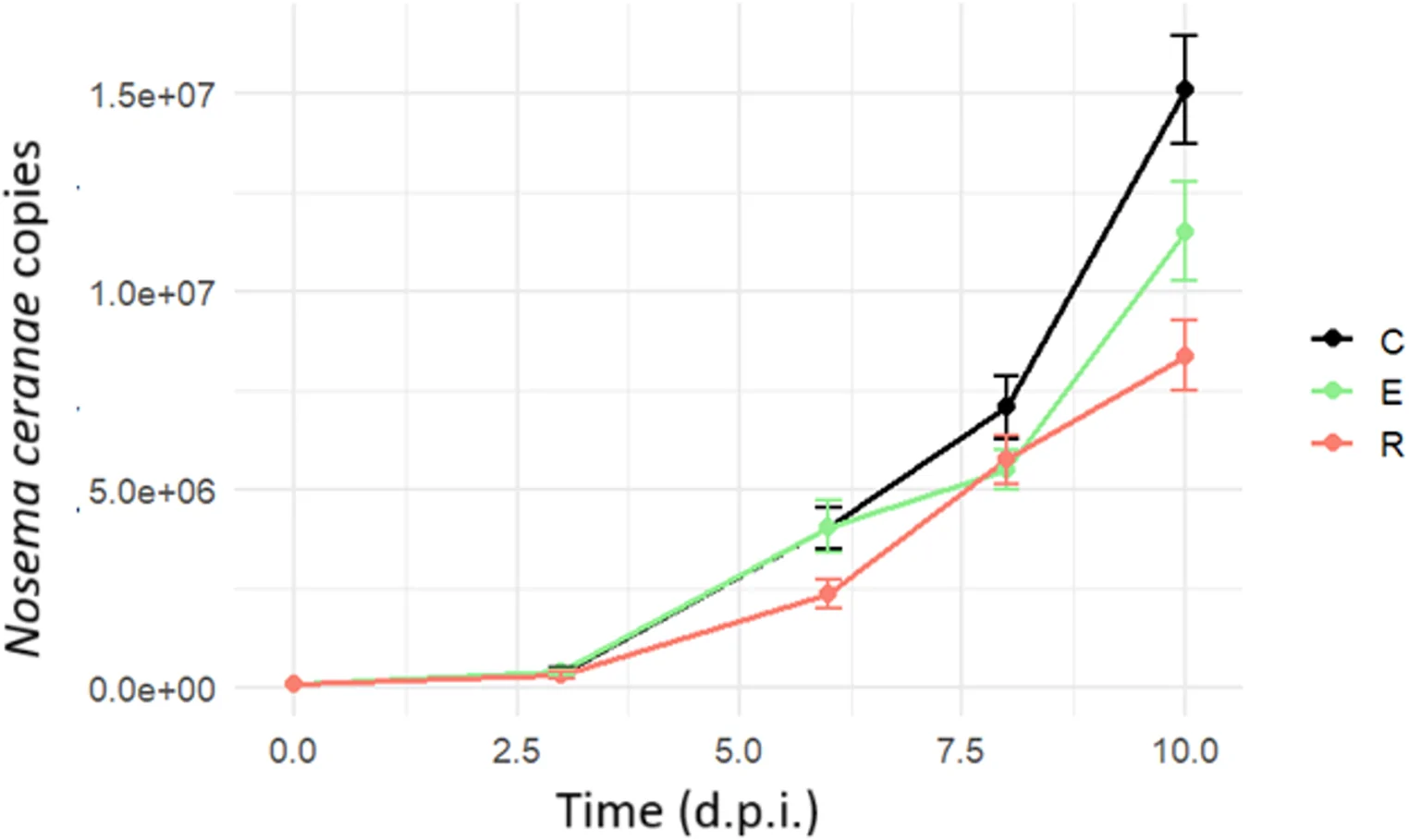
Variation in time (days post-infection, d.p.i.) of *Nosema ceranae* copy numbers in honey bees across treatment groups (C, E2, R2). Time 0 corresponds to infection inoculum spore level (100,000), added equally to all groups. Vertical bar indicate the standard error

*Effects of Treatments on Nosema ceranae Infection.* The results obtained for the log_10_-transformed *N. ceranae* copy numbers detected by qPCR determined overtime in the three treatment groups are reported in Fig. 7.

The analysis revealed a statistically significant effect of treatment (*F*(2,167) = 3.64, *P* < 0.05) and of time (*F*(1,167) = 461.94, *P* < 0.001). Post-hoc comparisons among treatment levels in time showed that the R2 group had the lowest infection level, significantly different from C (*P* < 0.05), while the E2 group was not significantly different from either C or R2.

At the end of the trial E2 and R2 treatments determined a significant inhibition of *N. ceranae* load by 23.8 and 44.6% respectively, compared to C (C-R2 and C-E2, *P* < 0.05), while difference between treatment was not significant.

## Discussion

Defatted seed meals of *E. sativa* and *B. nigra*, biomasses notably concentrated in GSLs, were previously tested as food supplements to control bee *N. ceranae* infection (Nanetti et al. 2021b; Ugolini et al. 2021a). In the present study, flowers of *E. sativa* and *R. lutea* were selected for their distinctive GSL profile, and high total GSL concentrations were found too. In particular, the flowers of *R. lutea* exhibited total GSL content comparable to that of defatted seed meals used in agricultural applications (Ugolini et al. 2021b). By contrast, mature seeds of *R. lutea* have previously been found to contain very low levels of GSLs (Pagnotta et al. 2020).

The use of intact freeze-dried *E. sativa* and *R. lutea* flowers for formulation development was explored. MYR activity was detected in flowers of both species, therefore, enzyme heat-deactivation was attempted to prevent GSL hydrolysis in the presence of water and avoid the production of high concentrations of hydrolysis products, ITCs in particular, which may act as deterrent to bees and inhibit feeding (Borges et al. 2020). Autolysis experiments performed on non-deactivated and thermally deactivated material showed the production of ITCs and NITs, respectively, which largely reflected the floral GSL profiles, with some exceptions. In non-deactivated *E. sativa* flowers, SF was not the predominant hydrolysis product despite precursor GRA being the main GSL identified in the flowers, but since SF is considered a labile compound susceptible to thermal degradation during GC analysis (Bennett et al. 2002; Raffo et al. 2018; Fechner et al. 2018) its contribution to hydrolytic profile may have been underestimated. In thermally deactivated *E. sativa* flowers, no ITCs were detected, confirming complete MYR inactivation after only 5 min of autoclave treatment. In contrast, NITs production was observed, likely resulting from chemically promoted GSL degradation due to the combined effects of temperature and pressure (Hanschen et al. 2018). Overall, GSL analysis of deactivated flowers confirmed a substantial reduction in GSL content after treatment. In *R. lutea*, all ITCs corresponding to the identified GSLs were detected following autolysis of non-deactivated flowers, except for the ITC derived from GBS. Although GBS is typically hydrolysed by MYR into its corresponding ITC, this compound is chemically unstable and rapidly converts into products such as 3,3′-diindolylmethane, indole-3-carboxaldehyde, and ascorbigen, which are not detectable by GC–MS analysis (Karanikolopoulou et al. 2021). Unlike *E. sativa*, complete MYR deactivation in *R. lutea* required a longer treatment time (10 min), as autolysis of 5 min-treated flowers still produced the 2-(α-L-rhamnopyranosyloxy)BITC. The higher MYR activity observed in *R. lutea* flowers may explain this behavior. As in *E. sativa* flowers, autoclave treatment resulted in a substantial loss of GSL content. In both *E. sativa* and *R. lutea*, NIT identification was further confirmed by performing autolysis on non-thermally treated flowers in the presence of FeSO₄, which is known to shift MYR-mediated GSLs hydrolysis toward NITs production rather than ITCs (Wittstock and Burow 2010).

The obtained results thus showed that the direct use of the flower powder is prevented by MYR activity, which in turn cannot be thermally deactivated without a great loss in GSLs.

The production of flower extracts overcame these issues and, furthermore, offered the advantage of concentrating bioactive compounds, such as GSLs, in a water-soluble powder, allowing their incorporation in sugar syrup formulations. The predominant GSLs were GRA and dimeric 4-MB GSL in *E. sativa*, and 2-(α-L- rhamnopyranosyloxyl) benzyl GSL in *R. lutea* extracts, respectively. Some authors speculated that dimeric 4-MB GSL is formed by non-enzymatic oxidation of 4-mercaptobutyl-GSL (4-MB GSL) in the extraction and purification process (Bennett et al. 2002). However, Cataldi et al. showed, by preventing oxidation throughout derivatization of the sulfur in the GSL side chain, that the disulfide is a naturally occurring GLS in *E. sativa* leaves (Cataldi et al. 2007).

The palatability and tolerability experiments showed that the syrup consumption by newly emerged bees was negatively affected by the presence of the extracts and by increasing concentrations. Extracts also caused a concentrations dependent reduction in bee survival compared to control, which was more pronounced for R2 compared to E2, while no significant difference between the E4 and R4 was observed. The lowest concentration of both extracts, E2 and R2, when administered to six-day-old bees artificially infected with *N. ceranae* spores, exhibited food consumption comparable to the control sugar syrup, although a slight decrease in bee survival was still observed. The different age of bees between the two trials could have affected their acceptance and tolerance of the extracts. Moreover, at the same lower concentration, both treatments showed a significant inhibitory effect on *N. ceranae* abundance development in bees compared to control, while no difference was found between the two extracts. It should be noted that, in other studies, although plant extracts tested against *N. ceranae* infection showed high toxicity to bees in laboratory cages, they did not produce negative effects if tested at the whole-colony level (Chaimanee et al. 2021; Ugolini et al. 2021a).

The results demonstrate that bees can metabolize floral GSLs into ITCs as their adducts were detected in gut tissues collected from the palatability and tolerability trials. Detectable amounts were found after two days of feeding, indicating a relatively rapid metabolism of ingested GSLs. ITC concentrations were influenced by multiple interacting factors, and they tended to accumulate in the hindgut, while ITC derived from *R. lutea* were generally higher than those from *E. sativa*, particularly over longer feeding periods. This indicated the presence of a MYR-like enzyme capable of hydrolyzing GSLs into ITCs as previously observed (Ugolini et al. 2021a). The origin of this MYR-like enzyme—whether it is part of the bee’s endogenous β-glucosidase system or derived from the gut microbiota—is still unknown. The higher levels of ITCs from *R. lutea* could be due to greater ITC stability, higher MYR-like enzyme affinity, or reduced metabolism by bee detoxification enzymes. Accumulation in the hindgut is partly expected as caged bees did not defecate during the trial, and confirmed results obtained in previous studies (Nanetti et al. 2021b; Ugolini et al. 2021a). ITCs, thus produced at the site of infection, could be considered the main contributors to the inhibitory activity of the extracts given their well-known antimicrobial properties, however, the contribution of other compounds cannot be excluded. Borges et al. tested D, L-SF, the hydrolysis product of the main GSL in *E. sativa* extract, as pure compound in laboratory trials on artificially infected bees and observed a strong inhibitory activity against *N. ceranae* (64% abundance reduction with 0.316 µmol/bee of SF over a period of 16 d.p.i.), but also a high toxicity, with reduced bee survival at high concentrations (Borges et al. 2020). The concentrations were higher than those used in this study, especially given the low GSL-to-ITC conversion rate observed when GSLs instead of pure ITCs are administered. Nanetti et al. evaluated candy supplemented with *E. sativa* defatted seed meals, in which the reduced form of GRA (GER) represented the main GSL, and reported about 50% inhibition at two concentrations (2 and 4% w/w) in similar trials (Nanetti et al. 2021b). However, in that study, *N. ceranae* abundance in control saples registered at the end of the trial was much lower than that obtained in the present work. These findings highlight how formulation, GSL profile or infection load can strongly influence the efficacy of the tested molecules. To the best of our knowledge, no studies have yet investigated the antimicrobial properties of dimeric 4-MB ITC detected in the floral *E. sativa* extract or 2-(α-L- rhamnopyranosyloxyl) benzyl ITC derived from *R. lutea*, whereas the antimicrobial activity of SF has been far more extensively studied (Moon et al. 2010; Krause et al. 2021). Its activity is thought to involve disruption of pathogen membrane integrity and activation of the ROS signaling pathway (He et al. 2024). Antibacterial, antioxidant and cytotoxic activities of *R. lutea* extracts or essential oil have been described by other authors (Sales et al. 2017; Asadi-Samani et al. 2019; Kiziltaş 2022). Alkaloids, flavonoids, saponins, anthocyanin, glucosides and tannins are considered active compounds contributing to the biological effects of *R. lutea* (Ali Esmail Al-Snafi 2022).

This is the first study investigating the potential of *E. sativa* and *R. lutea* flowers and the development of formulations rich in bioactive compounds for the containment of bee pathogens and, namely, of *N. ceranae*, as a safer and more sustainable alternative to conventional antibiotics. These products have the potential to protect bee health, especially if employed within an integrated management framework aimed at reducing the emergence of treatment resistance. Furthermore, like other plant extracts, they can be incorporated into feed or sugar syrups, making their administration practical and compatible with standard beekeeping practices. The development of such natural formulations represents a promising strategy to support colony health and productivity while aligning with ecological and food safety considerations. Many aspects, however, need further investigation as the anti-*N. ceranae* activity under laboratory conditions was accompanied by an evident fitness cost for the host, indicating that the tested doses may approach the upper limit of bee tolerance. Testing lower concentrations, mixing extracts from different species and try different administration timings, for example, may help improving efficacy while minimizing adverse effects on bee survival. Moreover, future studies should be conducted in field conditions to evaluate the effect of these extracts within the hive superorganism.

## Supplementary Information

Below is the link to the electronic supplementary material.

Supplementary Material 1 (PDF 625 KB)

## Data Availability

The data that support the findings of this study are available from the corresponding author upon reasonable request.

## References

[CR70] Agricolae (2006) Statistical Procedures for Agricultural Research. Contributed Packages, CRAN

[CR1] Ali Esmail Al-Snafi (2022) Constituents and biological effects of *Reseda lutea* and *Reseda odorata* grown in Iraq. Int J Biol Pharm Sci Archive 3:056–063. 10.53771/ijbpsa.2022.3.1.0031

[CR2] Arredondo D, Añón G, Campá J et al (2025) Propolis can reduce *Nosema ceranae* infection and enhance the immune response in honey bees, without disrupting the gut microbiota. J Invertebr Pathol 211:108333. 10.1016/j.jip.2025.108333 40221129

[CR3] Asadi-Samani M, Khaledi M, Khaledi F et al (2019) Phytochemical properties and antibacterial effects of *Salvia multicaulis* Vahl., *Euphorbia microsciadia* Boiss., and *Reseda lutea* on *Staphylococcus aureus* and *Acinetobacter baumanii*. Jundishapur J Nat Pharm Prod 14. 10.5812/jjnpp.63640

[CR4] Balfour NJ, Fensome KA, Samuelson EEW, Ratnieks FLW (2015) Following the dance: ground survey of flowers and flower-visiting insects in a summer foraging hotspot identified via honey bee waggle dance decoding. Agric Ecosyst Environ 213:265–271. 10.1016/j.agee.2015.08.007

[CR5] Bennett RN, Mellon FA, Botting NP et al (2002) Identification of the major glucosinolate (4-mercaptobutyl glucosinolate) in leaves of *Eruca sativa* L. (salad rocket). Phytochemistry 61:25–30. 10.1016/S0031-9422(02)00203-0 12165298

[CR6] Beran F, Pauchet Y, Kunert G et al (2014) *Phyllotreta striolata* flea beetles use host plant defense compounds to create their own glucosinolate-myrosinase system. Proc Natl Acad Sci 111. 10.1073/pnas.1321781111 PMC4034198 24799680

[CR7] Blažević I, Montaut S, Burčul F, Rollin P (2017) Glucosinolates: novel sources and biological potential pp 3–60

[CR8] Boonmee T, Sinpoo C, Laokulsiri K et al (2025) The in vitro potential of non-thermal atmospheric pressure plasma against *Nosema ceranae* infection in honeybees (*Apis mellifera*). Sci Rep 15:26975. 10.1038/s41598-025-11303-4 PMC12290067 40707618

[CR9] Borges D, Guzman-Novoa E, Goodwin PH (2020) Control of the microsporidian parasite *Nosema ceranae* in honey bees (*Apis mellifera*) using nutraceutical and immuno-stimulatory compounds. PLoS ONE 15:e0227484. 10.1371/journal.pone.0227484 PMC6953808 31923212

[CR10] Botías C, Martín-Hernández R, Meana A, Higes M (2013) Screening alternative therapies to control Nosemosis type C in honey bee (*Apis mellifera iberiensis*) colonies. Res Vet Sci 95:1041–1045. 10.1016/j.rvsc.2013.09.012 24148868

[CR11] Bremer PL, Vaniya A, Kind T et al (2022) How well can we predict mass spectra from structures? Benchmarking competitive fragmentation modeling for metabolite identification on untrained tandem mass spectra. J Chem Inf Model 62:4049–4056. 10.1021/acs.jcim.2c00936 PMC11521105 36043939

[CR12] Cataldi TRI, Rubino A, Lelario F, Bufo SA (2007) Naturally occurring glucosinolates in plant extracts of rocket salad (*Eruca sativa* L.) identified by liquid chromatography coupled with negative ion electrospray ionization and quadrupole ion-trap mass spectrometry. Rapid Commun Mass Spectrom 21:2374–2388. 10.1002/rcm.3101 17590871

[CR13] Cerny MS, Taube E, Battaglia R (1996) Identification of bis(4-isothiocyanatobutyl) disulfide and its precursor from rocket salad (*Eruca sativa*). J Agric Food Chem 44:3835–3839. 10.1021/jf960361r

[CR14] Česlová L, Klikarová J, Šalomounová T (2023) The content and profile of biologically active compounds present in individual parts of nasturtium (*Tropaeolum majus* L.): comprehensive study. Eur Food Res Technol 249:413–428. 10.1007/s00217-022-04126-4

[CR15] Chaimanee V, Kasem A, Nuanjohn T et al (2021) Natural extracts as potential control agents for *Nosema ceranae* infection in honeybees, *Apis mellifera*. J Invertebr Pathol 186:107688. 10.1016/j.jip.2021.107688 34728218

[CR16] Chiang WCK, Pusateri DJ, Leitz REA (1998) Gas chromatography/mass spectrometry method for the determination of sulforaphane and sulforaphane nitrile in Broccoli. J Agric Food Chem 46:1018–1021. 10.1021/jf970572b

[CR17] Cilia G, Cabbri R, Maiorana G et al (2018) A novel TaqMan ^®^ assay for *Nosema ceranae* quantification in honey bee, based on the protein coding gene Hsp70. Eur J Protistol 63:44–50. 10.1016/j.ejop.2018.01.007 29459253

[CR19] Cilia G, Sagona S, Giusti M et al (2019) *Nosema ceranae* infection in honeybee samples from Tuscanian Archipelago (Central Italy) investigated by two qPCR methods. Saudi J Biol Sci 26:1553–1556. 10.1016/j.sjbs.2018.11.017 PMC6864192 31762625

[CR18] Cilia G, Garrido C, Bonetto M et al (2020) Effect of Api-Bioxal^®^ and ApiHerb^®^ treatments against *Nosema ceranae* infection in *Apis mellifera* investigated by two qPCR methods. Vet Sci 7:125. 10.3390/vetsci7030125 PMC7558000 32899611

[CR20] Citi V, Piragine E, Pagnotta E et al (2019) Anticancer properties of erucin, an H_2_S-releasing isothiocyanate, on human pancreatic adenocarcinoma cells (AsPC-1). Phytother Res 33:845–855. 10.1002/ptr.6278 30632211

[CR52] R Core Team (2025) Version 4.5.1; R: A language and environment for statistical computing. R Foundation for Statistical Computing, Vienna, Austria. URL: https://www.R-project.org/

[CR21] Dufour V, Stahl M, Baysse C (2015) The antibacterial properties of isothiocyanates. Microbiol (N Y) 161. 10.1099/mic.0.082362-0 25378563

[CR22] Fechner J, Kaufmann M, Herz C et al (2018) The major glucosinolate hydrolysis product in rocket (*Eruca sativa* L.), sativin, is 1,3-thiazepane-2-thione: Elucidation of structure, bioactivity, and stability compared to other rocket isothiocyanates. Food Chem 261:57–65. 10.1016/j.foodchem.2018.04.023 29739606

[CR23] Finiguerra MG, Iori R, Palmieri S (2001) Soluble and total myrosinase activity in defatted *Crambe abyssinica* Meal. J Agric Food Chem 49:840–845. 10.1021/jf000917h 11262038

[CR24] Formato G, Rivera-Gomis J, Bubnic J et al (2022) Nosemosis prevention and control. Appl Sci 12:783. 10.3390/app12020783

[CR25] Franco P, Spinozzi S, Pagnotta E et al (2016) Development of a liquid chromatography–electrospray ionization–tandem mass spectrometry method for the simultaneous analysis of intact glucosinolates and isothiocyanates in Brassicaceae seeds and functional foods. J Chromatogr A 1428. 10.1016/j.chroma.2015.09.001 26363943

[CR26] Fulvio F, Zavatta L, Tiritelli R et al (2025) Impact of *Cannabis sativa* (L.) inflorescences on the control of artificial *Nosema* (= *Vairimorpha*) *ceranae* infection in honey bees *Apis mellifera**ligustica* (Spinola, 1806). Apidologie 56:83. 10.1007/s13592-025-01216-6

[CR27] Galletti S, Bernardi R, Leoni O et al (2001) Preparation and biological activity of four epiprogoitrin myrosinase-derived products. J Agric Food Chem 49. 10.1021/jf000736f 11170613

[CR28] Gambari L, Pagnotta E, Ugolini L et al (2024) Insights into osteogenesis induced by crude Brassicaceae seeds extracts: a role for glucosinolates. Nutrients 16:3457. 10.3390/nu16203457 PMC11510261 39458452

[CR29] Garrido PM, Porrini MP, Alberoni D et al (2024) Beneficial bacteria and plant extracts promote honey bee health and reduce *Nosema ceranae* infection. Probiotics Antimicrob Proteins 16:259–274. 10.1007/s12602-022-10025-7 PMC10850026 36637793

[CR30] Hanschen FS, Kühn C, Nickel M et al (2018) Leaching and degradation kinetics of glucosinolates during boiling of *Brassica oleracea* vegetables and the formation of their breakdown products. Food Chem 263:240–250. 10.1016/j.foodchem.2018.04.069 29784313

[CR31] He L, Jiang H, Li Y et al (2024) Sulforaphane-enriched extracts from Broccoli exhibit antimicrobial activity against plant pathogens, promising a natural antimicrobial agent for crop protection. Biomolecules 14:352. 10.3390/biom14030352 PMC10968597 38540770

[CR32] Higes M, Martín-Hernández R, Meana A (2010) *Nosema ceranae* in Europe: an emergent type C nosemosis. Apidologie 41:375–392. 10.1051/apido/2010019

[CR33] Iorizzo M, Letizia F, Ganassi S et al (2022) Recent advances in the biocontrol of nosemosis in honey bees (*Apis mellifera* L). J Fungi 8:424. 10.3390/jof8050424 PMC9145624 35628680

[CR34] ISO9167-1 :1992/Amd 1:2013 (1992) Graines de colza—dosage des glucosinolates—Partie 1: methode par chromatographie liquide à haute performance. Geneva, Switzerland

[CR35] Jabal-Uriel C, Alba C, Higes M et al (2022) Effect of *Nosema ceranae* infection and season on the gut bacteriome composition of the European honeybee (*Apis mellifera*). Sci Rep 12:9326. 10.1038/s41598-022-13337-4 PMC9167302 35662256

[CR36] Karanikolopoulou S, Revelou P-K, Xagoraris M et al (2021) Current methods for the extraction and analysis of isothiocyanates and indoles in Cruciferous vegetables. Analytica 2:93–120. 10.3390/analytica2040011

[CR37] Kassambara A, Kosinski M, Biecek P (2016) survminer: Drawing Survival Curves using ggplot2. CRAN. Contributed Packages

[CR38] Kiziltaş H (2022) Comprehensive evaluation of *Reseda lutea* L. (Wild Mignonette) and 7 isolated flavonol glycosides: determination of antioxidant activity, anti-Alzheimer, antidiabetic and cytotoxic effects with in vitro and in silico methods. Turk J Chem 46:1185–1198. 10.55730/1300-0527.3426 PMC10395699 37538778

[CR39] Krause K, Pyrczak-Felczykowska A, Karczewska M et al (2021) Dietary isothiocyanates, sulforaphane and 2-phenethyl isothiocyanate, effectively impair *Vibrio cholerae* Virulence. Int J Mol Sci 22:10187. 10.3390/ijms221910187 PMC8508596 34638525

[CR40] Moon J-K, Kim J-R, Ahn Y-J, Shibamoto T (2010) Analysis and anti-*Helicobacter* activity of sulforaphane and related compounds present in Broccoli (*Brassica oleracea* L.) sprouts. J Agric Food Chem 58:6672–6677. 10.1021/jf1003573 20459098

[CR42] Nanetti A, Rodriguez-García C, Meana A et al (2015) Effect of oxalic acid on *Nosema ceranae* infection. Res Vet Sci 102:167–172. 10.1016/j.rvsc.2015.08.003 26412538

[CR41] Nanetti A, Bortolotti L, Cilia G (2021a) Pathogens spillover from honey bees to other arthropods. Pathogens 10:1044. 10.3390/pathogens10081044 PMC8400633 34451508

[CR43] Nanetti A, Ugolini L, Cilia G et al (2021b) Seed meals from *Brassica nigra* and *Eruca sativa* control artificial *Nosema ceranae* infections in *Apis mellifera*. Microorganisms 9:949. 10.3390/microorganisms9050949 PMC8146933 33924845

[CR44] Narbad A, Rossiter JT (2018) Gut glucosinolate metabolism and isothiocyanate production. Mol Nutr Food Res 62. 10.1002/mnfr.201700991 PMC6767122 29806736

[CR45] Okumus E (2024) Effect of ultrasonic and conventional extraction on bioactive components, glucosinolate content and antidiabetic activity of *Crambe tataria*. Fitoterapia 178:106177. 10.1016/j.fitote.2024.106177 39122120

[CR46] Pagnotta E, Agerbirk N, Olsen CE et al (2017) Hydroxyl and methoxyl derivatives of benzylglucosinolate in *Lepidium densiflorum* with hydrolysis to isothiocyanates and non-isothiocyanate products: substitution governs product type and mass spectral fragmentation. J Agric Food Chem 65:3167–3178. 10.1021/acs.jafc.7b00529 28343387

[CR47] Pagnotta E, Montaut S, Matteo R et al (2020) Glucosinolates in *Reseda lutea* L.: Distribution in plant tissues during flowering time. Biochem Syst Ecol 90:104043. 10.1016/j.bse.2020.104043

[CR49] Pagnotta E, Ugolini L, Matteo R, Righetti L (2022) Bioactive compounds from *Eruca sativa* seeds. Encyclopedia 2:1866–1879. 10.3390/encyclopedia2040129

[CR48] Pagnotta E, Righetti L, Micheletti G et al (2025) Effect of sodium sulfate treatment on the modulation of aliphatic glucosinolates in *Eruca sativa* Mill organs at flowering stage. Appl Sci 15:8757. 10.3390/app15158757

[CR50] Pinheiro J, Bates D (1999) nlme: Linear and Nonlinear Mixed Effects Models. Contributed Packages, CRAN

[CR51] Porrini MP, Garrido PM, Umpiérrez ML et al (2020) Effects of synthetic acaricides and *Nosema ceranae* (Microsporidia: Nosematidae) on molecules associated with chemical communication and recognition in Honey Bees. Vet Sci 7:199. 10.3390/vetsci7040199 PMC7768465 33302502

[CR53] Radulović NS, Zlatković DB, Ilić-Tomić T et al (2014) Cytotoxic effect of *Reseda lutea* L.: A case of forgotten remedy. J Ethnopharmacol 153:125–132. 10.1016/j.jep.2014.01.034 24509155

[CR54] Raffo A, Masci M, Moneta E et al (2018) Characterization of volatiles and identification of odor-active compounds of rocket leaves. Food Chem 240:1161–1170. 10.1016/j.foodchem.2017.08.009 28946238

[CR55] Romeo L, Iori R, Rollin P et al (2018) Isothiocyanates: an overview of their antimicrobial activity against human infections. Molecules 23. 10.3390/molecules23030624 PMC6017699 29522501

[CR56] Sales AJ, Y B PM et al (2017) Evaluation of the antimicrobial effects of essential oil of *Reseda Lutea* L. on pathogenic bacteria: *Staphylococcus aureus*, *Staphylococcus epidermidis*, and *Escherichia coli*. Arch Clin Microbiol 08. 10.4172/1989-8436.100041

[CR57] Searle SR, Speed FM, Milliken GA (1980) Population Marginal Means in the Linear Model: An Alternative to Least Squares Means. Am Stat 34:216–221. 10.1080/00031305.1980.10483031

[CR58] Shakeel M, Ali H, Ahmad S et al (2019) Insect pollinators diversity and abundance in *Eruca sativa* Mill. (Arugula) and *Brassica rapa* L. (Field mustard) crops. Saudi J Biol Sci 26:1704–1709. 10.1016/j.sjbs.2018.08.012 PMC6864147 31762647

[CR59] Singh G, Rana A (2025) Honeybees and colony collapse disorder: understanding key drivers and economic implications. 91:750–766. 10.1007/s43538-025-00399-x

[CR60] Tache B, Spulber R, Dinu L-D, Vamanu E (2025) Exploring eco-friendly microbial strategies for nosemosis control in honeybee. Microorganisms 13:2357. 10.3390/microorganisms13102357 PMC12566385 41156816

[CR61] Therneau TM (2001) survival: Survival Analysis. CRAN: Contributed Packages

[CR62] Ugolini L, Cilia G, Pagnotta E et al (2021a) Glucosinolate bioactivation by *Apis mellifera* workers and its impact on *Nosema ceranae* infection at the colony level. Biomolecules 11:1657. 10.3390/biom11111657 PMC8615805 34827655

[CR63] Ugolini L, Scarafile D, Matteo R et al (2021b) Effect of bioactive compounds released from Brassicaceae defatted seed meals on bacterial load in pig manure. Environ Sci Pollut Res 28. 10.1007/s11356-021-14321-7 PMC8589757 34191264

[CR64] Urbieta-Magro A, Higes M, Meana A et al (2019) Age and method of inoculation influence the infection of worker honey Bees (*Apis mellifera*) by *Nosema ceranae*. Insects 10:417. 10.3390/insects10120417 PMC6956240 31766667

[CR65] Whatelet J-P, Iori R, Leoni O et al (2004) Guidelines for glucosinolate analysis in green tissues used for biofumigation. Agroindustria 257–266

[CR66] Wickham H (2016) Data Analysis. pp 189–201

[CR67] Wickham H, François R, Henry L et al (2014) dplyr: A Grammar of Data Manipulation. Contributed Packages, CRAN

[CR68] Wittstock U, Burow M (2007) Tipping the Scales - Specifier proteins in glucosinolate hydrolysis. IUBMB Life 59. 10.1080/15216540701736277 18085474

[CR69] Wittstock U, Burow M (2010) Glucosinolate breakdown in Arabidopsis: mechanism, regulation and biological significance. Arabidopsis Book 8:e0134. 10.1199/tab.0134 PMC3244901 22303260

