# Supplementary material for: Glucosinolate-Rich Floral Extracts Hinder Nosema (= Vairimorpha) ceranae Infection in Caged Apis mellifera Workers
Source: J Chem Ecol. 2026 Jul 9;52(4):63. doi: 10.1007/s10886-026-01736-0 (PMC13350151; doi:10.1007/s10886-026-01736-0)
Supplement: Supplementary file 1 — Supplementary Material 1 (PDF 625 KB) [file 10886_2026_1736_MOESM1_ESM.pdf]

Journal of Chemical Ecology

Glucosinolate-Rich Floral Extracts Hinder *Nosema* (=*Vairimorpha*) *ceranae* Infection in Caged *Apis Mellifera* Workers

MATTEO CARLONI[1-2], ELEONORA PAGNOTTA[2], LAURA RIGHETTI[2], LORENA MALAGUTI[2], VITTORIO CAPANO[3], ANTONIO NANETTI[3], NICOLA PECCHIONI[1-4], MANUELA BAGATTA[2, †], LUISA UGOLINI[2, *, †]

[1]*Department of Life Sciences, University of Modena and Reggio Emilia, Modena, Italy - Padiglione Besta, Via Amendola 2, 242122 Reggio Emilia – Italy*; Matteo Carloni, 320549@studenti.unimore.it, 0009-0004-9450-2673; Nicola Pecchioni, nicola.pecchioni@unimore.it, 0000-0003-1704-2541

[2]*Consiglio per la Ricerca in Agricoltura e l'Analisi dell'Economia Agraria (CREA), Centro di ricerca in Cerealicoltura e Colture Industriali, Via di Corticella 133, 40128, Bologna, Italy*; Eleonora Pagnotta, eleonora.pagnotta@crea.gov.it, 0000-0002-9477-1724; Laura Righetti, laura.righetti@crea.gov.it, 0000-0003-4238-0665; Lorena Malaguti, lorena.malaguti@crea.gov.it, 0009-0008-2812-2858; Manuela Bagatta, manuela.bagatta@crea.gov.it, 0000-0002-8928-4497; Luisa Ugolini, luisa.ugolini@crea.gov.it, 0000-0001-7954-6257

[3]*Consiglio per la Ricerca in Agricoltura e l'Analisi dell'Economia Agraria (CREA), Centro di ricerca in Agricoltura e Ambiente, Via di Corticella 133, 40128 Bologna, Italy*; Vittorio Capano, vittorio.capano@crea.gov.it, 0000-0003-2966-9819; Antonio Nanetti, antonio.nanetti@crea.gov.it, 0000-0002-1655-6754

[4]*Consiglio per la Ricerca in Agricoltura e l'Analisi dell'Economia Agraria (CREA), Centro di ricerca in Cerealicoltura e Colture Industriali, S.S. 673 metri 25, 200, 71122 Foggia, Italy*; Nicola Pecchioni, nicola.pecchioni@crea.gov.it, 0000-0003-1704-2541

*Corresponding author LUISA UGOLINI (e-mail: luisa.ugolini@crea.gov.it)

† These authors share last authorship

Figure S1. Representative HPLC-UV chromatograms of *Eruca sativa* flower (red line) and of *Reseda lutea* flower (blue line) desulfated GSLs. The main identified glucosinolates are indicated as follow: 1-glucoraphanin; 2 -dimeric 4-mercaptobutyl GSL; 3 – 3-hydroxybenzyl GSL; 4- benzyl GSL; 5 - 2-(α-L-rhamnopyranosyloxy)benzyl GSL; 6 –non GSL peak; 7 – glucobrassicin. IS, internal standard (sinigrin)

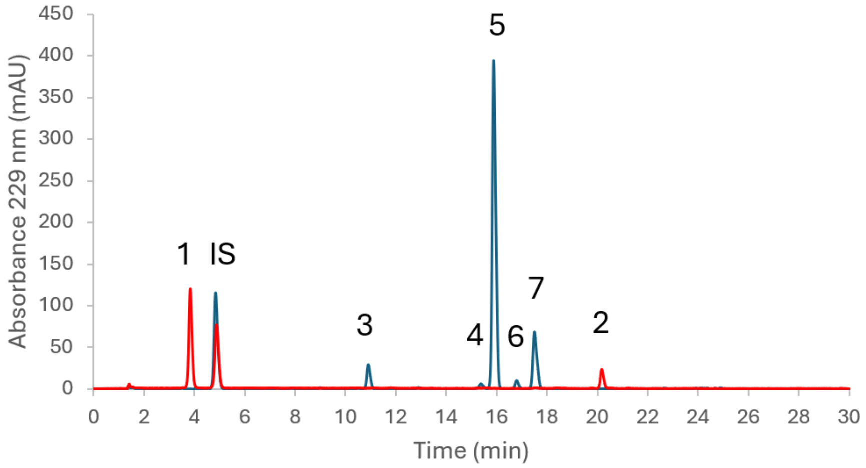

Figure S2. GC chromatograms of GSL hydrolysis products formed by *Eruca sativa* flowers autolysis conducted under different conditions and analyzed by GC-MS. Autolysis was performed on flower powders: A) not deactivated; B) not deactivated and in presence of tris(2-carboxyethyl) phosphine hydrochloride (TCEP); C) deactivated by autoclave treatment (5 min at 120 °C and 1 bar); D) not deactivated and in presence of $FeSO_4$. Glucoerucin (GER); sulforaphane (SF); 4-(mercaptobutyl) (4 MB), bis(4-isothiocyanatobutyl)disulphide (dimeric-4MB ITC), isothiocyanate (ITC), nitrile (NIT)

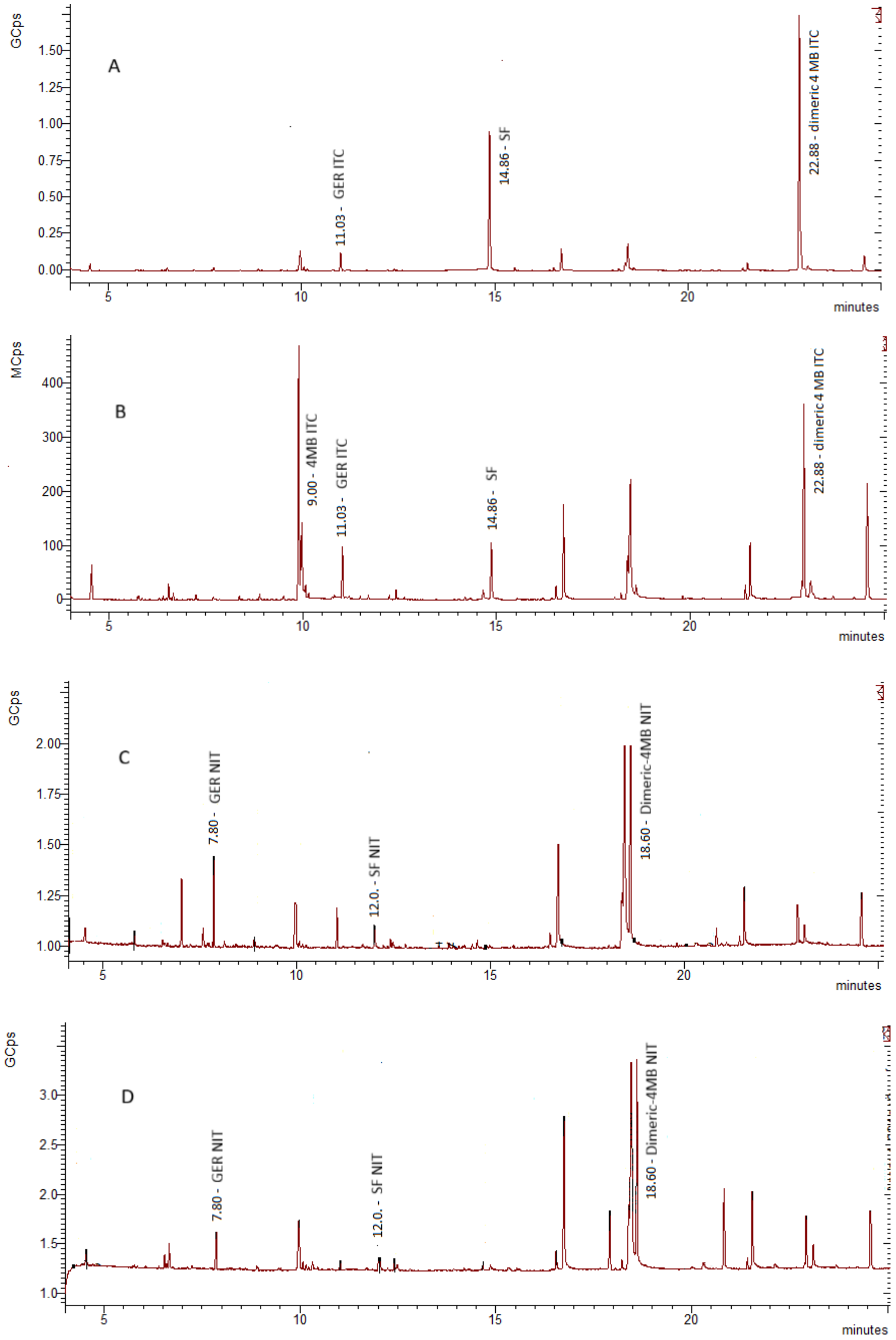

Figure S3. GSL hydrolysis products mass spectra formed from *Eruca sativa* flower autolysis. Glucoerucin (GER); sulforaphane (SF); (4-(mercaptobutyl) (4 MB), isothiocyanate (ITC), nitrile (NIT)

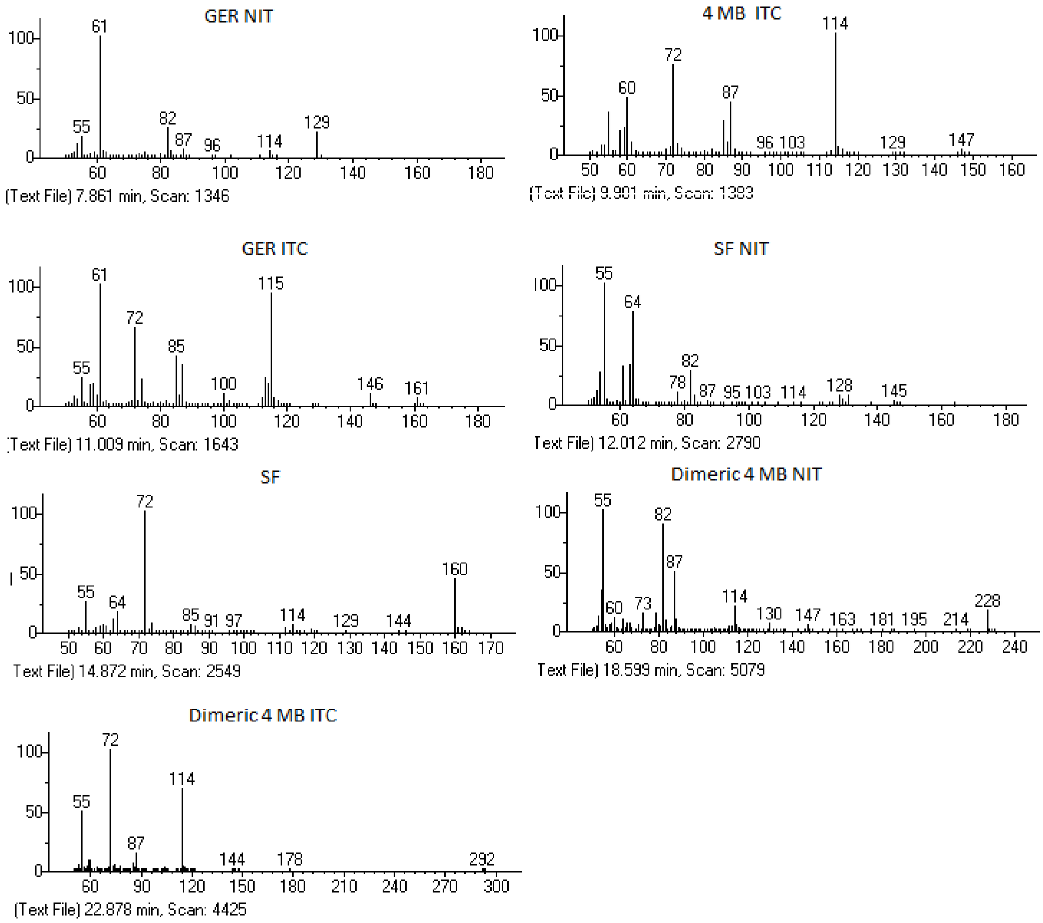

Figure S4. GC chromatograms of GSL hydrolysis products formed by *Reseda lutea* flowers autolysis conducted under different conditions and analyzed by GC-MS. Autolysis was performed on flower powders: A) not deactivated; B) and C) deactivated by autoclave treatment for 5 and 10 min at 120 °C and 1 bar, respectively; D) not deactivated and in presence of $FeSO_4$. Benzyl (B), isothiocyanate (ITC), nitrile (NIT), indol-3-ylmethyl- (GBS). 2-(α-L- rhamnopyranosyloxyl) (RAM)

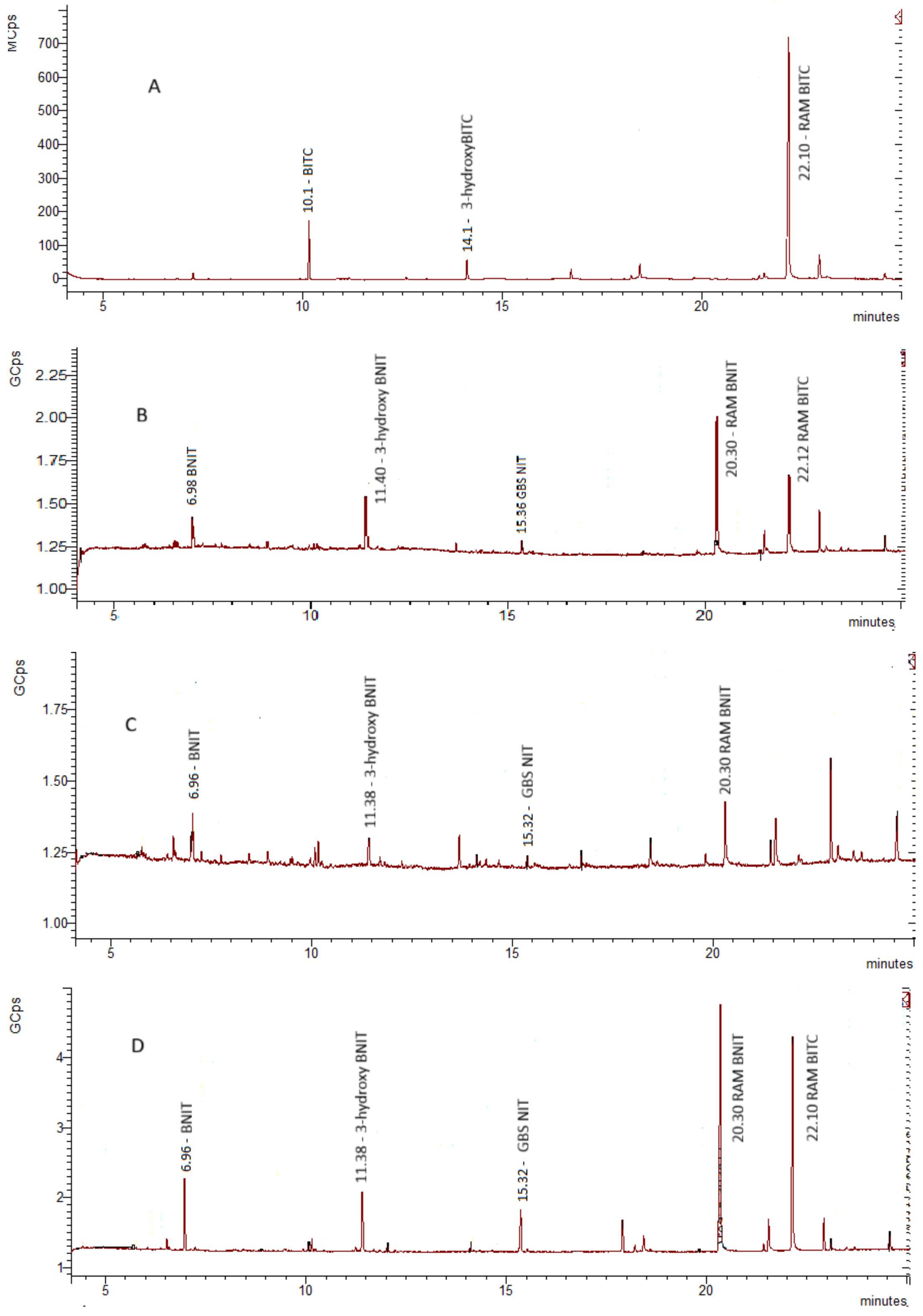

Figure S5. GSL hydrolysis products mass spectra formed from *Reseda Lutea* flower autolysis. Benzyl (B), isothiocyanate (ITC), nitrile (NIT), indol-3-ylmethyl- (GBS)

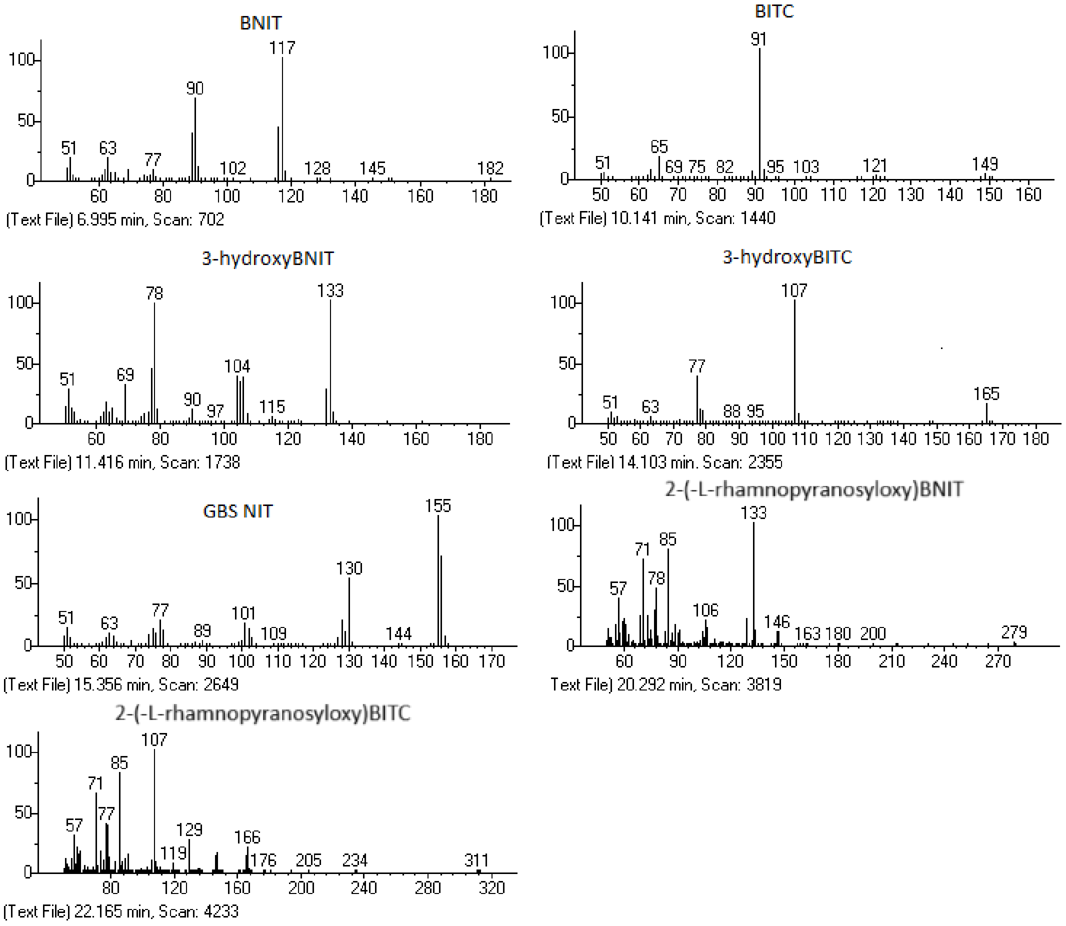

Table S1. Chromatogram retention time (t_R), mass spectra (MS) peak composition and identification methods (ID) of GSL hydrolysis products formed by *Eruca sativa* flowers autolysis. ID: STD (mass spectrum and t_R comparison with authentic standards); lib (NIST match); Ref (comparison with literature spectra); put (putative identification). Glucoerucin (GER); sulforaphane (SF); 4-(mercaptobutyl)isothiocyanate (4-MB ITC), 4-(mercaptobutyl)nitrile (4-MB NIT), bis(4-isothiocyanatobutyl)disulfide (dimeric 4-MB ITC), bis(cyanatobutyl)disulfide (dimeric 4-MB NIT), isothiocyanate (ITC), nitrile (NIT)

GC t_R (min)	Compounds	MS, 70 eV, m/z (rel int)	ID
7.9	GER NIT	55 (17), 61 (100), 82 (25), 129 (20) [M^+]	STD (Galletti et al. 2001)
9.9	4-MB ITC	55 (34), 60 (44), 72 (71), 87 (44), 114 (100); 147 [M^+]	Ref (Cerny et al. 1996; Bennett et al. 2002; Raffo et al. 2018; Fechner et al. 2018)
11.0	GER ITC	55(23), 61(100), 72 (65), 85 (41), 115 (92); 161 [M^+]	STD (Citi et al. 2019)
12.0	SF NIT	55 (100), 64 (62), 82 (27); 145 [M^+]	Lib + Ref (Chiang et al. 1998)
14.9	SF	55 (26), 64 (17), 72 (100), 114 (7), 160 (47); 177 [M^+]	Lib + Ref (Chiang et al. 1998)
18.6	Dimeric 4-MB NIT	55 (100), 82 (85), 87 (47), 114 (20), 228 [M^+]	Ref (Bennett et al. 2002)
22.9	Dimeric 4-MB ITC	55 (48), 72 (100), 87 (14), 114 (68); 292 [M^+]	Ref (Cerny et al. 1996)

Table S2 Chromatogram retention time (t_R), mass spectra (MS) peak composition and identification methods (ID) of GSL hydrolysis products formed by *reseda lutea* flowers autolysis. ID: STD, mass spectrum and t_R comparison with authentic standards; lib, NIST match; Ref, comparison with literature spectra; put, putative identification. Benzyl (B), isothiocyanate (ITC), nitrile (NIT), indol-3-ylmethyl- (GBS)

GC t_R (min)	Compounds	MS, 70 eV, m/z (rel int)	ID
7.0	BNIT	51 (19), 63 (18), 90 (66), 117 (100) [M^+]	STD[1]
10.1	BITC	65 (17), 91 (100), 149 (5) [M^+]	STD[1]
11.4	3-hydroxyBNIT	51 (27), 63 (17), 78 (97), 104 (38), 133 (100) [M^+]	Put (Bremer et al. 2022)
14.1	3-hydroxyBITC	51 (8), 77 (4), 107 (100), 165 (16) [M^+]	Put (Bremer et al. 2022)
15.3	GBS NIT	51 (14), 77(19), 101 (17), 130 (52), 155 (100); 156 [M^+]	Lib + Ref (Songsak and Lockwood 2004)
20.3	2-(α-L- rhamnopyranosyloxyl)BNIT	57 (39), 71 (70), 78 (47), 85 (78), 106 (23), 129 (22), 133 (100), 279 [M^+]	Put (Bremer et al. 2022)
22.1	2-(α-L- rhamnopyranosyloxyl)BITC	57 (33), 71 (70), 77 (42), 85 (81), 107 (100), 129 (27), 166 (21); 311 [M^+]	Ref (Radulović et al. 2014)

[1]Sigma Aldrich

Table S3. *Eruca sativa* glucosinolate (GSL) stability in the extract dissolved in diluted sugar syrup at two doses, 2 and 4 μmol g^{-1}, and incubated at 33°C for 0 and 3 days (time). GSL means concentration of three replicates are expressed as μmol g^{-1} ± standard deviation, and the residual % compared to time 0 is reported (%)

Dose	GSL	Time	μmol g^{-1}	%
2	GRA	0	69.1 ± 11.1	100
		3	66.6 ± 1.2	96
	GER	0	2.7 ± 0.6	100
		3	2.5 ± 0.2	91
	Dimeric-4MB GSL	0	26.2 ± 2.2	100
		3	25.3 ± 2.1	97
	Total	0	98.0 ± 9.5	100
		3	94.4 ± 1.0	96
4	GRA	0	67.2 ± 0.4	100
		3	70.2 ±0.8	104
	GER	0	2.4 ±0.1	100
		3	2.6 ±0.1	110
	Dimeric-4MB GSL	0	30.7 ± 0.2	100
		3	28.4 ± 0.5	92
	Total	0	100.3 ± 0.6	100
		3	101.2 ± 1.3	101

Table S4. *Reseda lutea* glucosinolate (GSL) stability in the extract dissolved in diluted sugar syrup at two doses, 2 and 4 μmol g^{-1}, and incubated at 33°C for 0 and 3 days (time). GSL means concentration of three replicates are expressed as μmol g^{-1} ± standard deviation and the residual % compared to time 0 is reported (%)

Dose	GSL	Time	μmol g^{-1}	%
2	Benzyl GSL	0	8.9 ± 0.4	100
		3	10.8 ± 1.7	122
	2-(α-L- rhamnopyranosyloxyl) benzyl GSL	0	142.8 ± 0.8	100
		3	147.6 ± 2.1	103
	GBS	0	13.8 ± 0.4	100
		3	14.0 ± 0.1	101
	3-hydroxybenzyl GSL	0	19.5 ±0.3	100
		3	19.6 ±0.5	100
	Total	0	184.5 ± 5.9	100
		3	192.1 ± 1.0	101
4	Benzyl GSL	0	11.6 ± 1.1	100
		3	9.2 ± 0.7	79
	2-(α-L- rhamnopyranosyloxyl) benzyl GSL	0	142.4 ± 1.3	100
		3	154 ± 9.3	108
	GBS	0	13.8 ± 0.1	100
		3	14.2 ± 0.9	103
	3-hydroxybenzyl GSL	0	18.9 ± 0.1	100
		3	20.6 ±1.3	109
	Total	0	186.7 ± 2.4	100
		3	198 ±12.2	106

Table S5. Palatability and tolerability trial survival analysis. Number of observations (records), number of deaths (events), restricted mean survival time (rmean) ± standard error (SE); lower and upper limits of the 95% confidence interval for the rmean, median survival time, time when 50% of a population is still alive (median) for the treated (E2, E4, R2, R4) and control (C) groups

Group	records	events	Rmean ± SE	0.95 C.I.	median
C	122	88	26.72133 ± 0.587677	25.56948-27.87318	27
E2	117	83	18.26025 ± 0.327494	17.61836-18.90214	20
E4	121	87	13.20475 ± 0.287839	12.64059-13.76892	14
R2	123	90	15.627 ± 0.376702	14.88866-16.36533	16
R4	121	88	13.17533 ± 0.242391	12.70024-13.65042	13

Table S6. *Nosema ceranae* trial survival analysis. Number of observations (records), number of deaths (events), restricted mean survival time (rmean) ± standard error (SE); lower and upper limits of the 95% confidence interval for the rmean, median survival time, time when 50% of a population is still alive (median) for the treated (E2, R2) and control (C) groups

Group	records	events	Rmean ± SE	0.95 C.I.	median
C	90	12	14.47177 ± 0.5743864	13.34597-15.59757	15
E2	96	34	11.54423 ± 0.4248717	10.71148-12.37698	13
R2	92	31	11.20244 ± 0.4378334	10.34429-12.06059	13

REFERENCES

Bennett RN, Mellon FA, Botting NP, et al (2002) Identification of the major glucosinolate (4-mercaptobutyl glucosinolate) in leaves of Eruca sativa L. (salad rocket). Phytochemistry 61:25–30. https://doi.org/10.1016/S0031-9422(02)00203-0

Bremer PL, Vaniya A, Kind T, et al (2022) How Well Can We Predict Mass Spectra from Structures? Benchmarking Competitive Fragmentation Modeling for Metabolite Identification on Untrained Tandem Mass Spectra. J Chem Inf Model 62:4049–4056. https://doi.org/10.1021/acs.jcim.2c00936

Cerny MS, Taube E, Battaglia R (1996) Identification of Bis(4-isothiocyanatobutyl) Disulfide and Its Precursor from Rocket Salad (*Eruca sativa*). J Agric Food Chem 44:3835–3839. https://doi.org/10.1021/jf960361r

Chiang WCK, Pusateri DJ, Leitz REA (1998) Gas Chromatography/Mass Spectrometry Method for the Determination of Sulforaphane and Sulforaphane Nitrile in Broccoli. J Agric Food Chem 46:1018–1021. https://doi.org/10.1021/jf970572b

Citi V, Piragine E, Pagnotta E, et al (2019) Anticancer properties of erucin, an H2S-releasing isothiocyanate, on human pancreatic adenocarcinoma cells (AsPC-1). Phytotherapy Research 33:845–855. https://doi.org/https://doi.org/10.1002/ptr.6278

Fechner J, Kaufmann M, Herz C, et al (2018) The major glucosinolate hydrolysis product in rocket (Eruca sativa L.), sativin, is 1,3-thiazepane-2-thione: Elucidation of structure, bioactivity, and stability compared to other rocket isothiocyanates. Food Chem 261:57–65. https://doi.org/10.1016/j.foodchem.2018.04.023

Galletti S, Bernardi R, Leoni O, et al (2001) Preparation and Biological Activity of Four Epiprogoitrin Myrosinase-Derived Products. J Agric Food Chem 49:. https://doi.org/10.1021/jf000736f

Radulović NS, Zlatković DB, Ilić-Tomić T, et al (2014) Cytotoxic effect of Reseda lutea L.: A case of forgotten remedy. J Ethnopharmacol 153:125–132. https://doi.org/10.1016/j.jep.2014.01.034

Raffo A, Masci M, Moneta E, et al (2018) Characterization of volatiles and identification of odor-active compounds of rocket leaves. Food Chem 240:1161–1170. https://doi.org/10.1016/j.foodchem.2017.08.009

Songsak T, Lockwood GB (2004) Production of two volatile glucosinolate hydrolysis compounds in Nasturtium montanum and Cleome chelidonii plant cell cultures. Fitoterapia 75:296–301. https://doi.org/10.1016/j.fitote.2004.01.007
